# Breaking Bonds at Tin(II): Reductive or Oxidative Addition?

**DOI:** 10.1002/anie.202503050

**Published:** 2025-06-12

**Authors:** Maximilian Dietz, Josef T. Boronski, Amelia M. Swarbrook, Simon Aldridge

**Affiliations:** ^1^ Inorganic Chemistry Laboratory, Department of Chemistry University of Oxford South Parks Road Oxford OX1 3QR UK; ^2^ Department of Chemistry, Molecular Sciences Research Hub Imperial College London 82 Wood Lane White City London W12 0BZ UK

**Keywords:** Beryllium, Borazine, Boron, Main group, Organometallics, Tin

## Abstract

Here, the addition of H─H, Be─Be, B─B, and B─H bonds to Sn^II^ is studied by experimental and theoretical means. We report the first examples of B─B and Be─Be bond additions to a main group metal centre, along with the first example of borazine B─H bond addition to any element of the Periodic Table. Quantum chemical calculations suggest that the classification of these reactions as prototypical “oxidative” addition processes may be misleading and that a more nuanced description is warranted.

## Graphical abstract

Typically, the addition of a chemical bond to a metal centre is assumed─without question─to be an oxidative process. Here, through studies of bond addition to tin(II), we reveal a spectrum ranging from ‘oxidative’ (Cl─Cl) to ‘reductive’ (Be─Be) processes, with other bonds (B─B, B─H, H─H) falling between these two extremes.

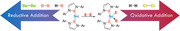

The addition of element‐element (E–E’) bonds to metal (M) centres is key to a range of industrially critical chemical transformations.^[^ ^1^ ^]^ For example, the addition of H─H, B─B, and B─H bonds to *d*‐block metal centres has been studied extensively due to the relevance of these reactions to hydrogenation and borylation catalysis, respectively.^[^ ^2^, ^3^, ^4^, ^5^ ^]^ In more general terms, whether such bond addition processes at M are *formally* “oxidative” or “reductive” will depend on the identity of M, E, and E’ and their respective electronegativities.^[^ ^6^, ^7^, ^8^ ^]^ Although this may seem a simple taxonomic matter, the electro‐ or nucleophilic character of the three elemental centres in the resulting M(E)(E’) complex has profound implications for their further reactivity, and consequently for synthetic applications of these species.^[^ ^9^, ^10^, ^11^ ^]^ Furthermore, the influence(s) of the ancillary ligands at M, as well as similarities between the electronegativity of E and M, can lead to ambiguity regarding the polarity of the M–E/E’ bond.^[^ ^12^, ^13^, ^14^, ^15^, ^16^, ^17^, ^18^ ^]^

The transition metal‐like reactivity of main group elements has received a great deal of attention in recent decades.^[^ ^19^, ^20^, ^21^, ^22^, ^23^, ^24^, ^25^ ^]^ Indeed, the “oxidative addition” of E–E’ bonds by complexes that feature low‐oxidation‐state main group centres is now relatively well‐developed.^[^ ^26^ ^]^ However, the addition of a B─B bond to a single main group metal site—a reaction of relevance to potential main group‐mediated diboration reactions—has not yet been demonstrated.^[^ ^26^, ^27^, ^28^, ^29^, ^30^ ^]^ Analogously, the addition of the Be─Be bonds of diberyllanes at main group centres also remains to be reported.^[^ ^31^, ^32^, ^33^, ^34^, ^35^ ^]^ One remarkably reactive low‐oxidation state main group complex is the bis(boryl)stannylene Sn[B]_2_ (**1**, where [B] = B(NDippCH)_2_, Dipp = 2,6‐*^i^* Pr_2_C_6_H_3_), which has an extremely small HOMO‐LUMO energy separation, principally due to the potent σ‐donor properties of the boryl ancillary ligands.^[^ ^36^, ^37^, ^38^, ^39^ ^]^ Consequently, **1** has been shown to oxidatively add a range of hetero‐elemental E–H bonds (E = N, O, B, Si), as well as the homo‐elemental H─H bond of dihydrogen.^[^ ^37^ ^]^ To complement H─H bond activation chemistry, we were interested in examining whether the addition of Be─Be and B─B bonds at Sn was possible, with a view to studying and rationalising the differences in the electronic structure of the tin centre across the resulting series of complexes.

Here, we describe the addition of B─B and Be─Be bonds to Sn—the first examples of the addition of these linkages at a single main group metal centre. Furthermore, we report borazine B–H bond addition to Sn—a reaction which represents the first example of the addition of this linkage to any element.^[^ ^40^, ^41^, ^42^, ^43^ ^]^ Although the insertion of Sn^II^ into H─H, B─B, and B─H bonds would be classed as an “oxidative” addition based on Pauling electronegativities, our quantum chemical calculations suggest that this formalism may not accurately reflect the true character of this redox process.^[^ ^6^ ^]^ Therefore, we propose that the nature of an addition reaction can be considered to lie on a spectrum from “oxidative” to “reductive”, with many bond addition processes sitting between these two extremes. This observation has implications for the design of functional complexes featuring elements from across the Periodic Table.

Reaction of Sn[B]_2_ (**1**; [B] = B(NDippCH)_2_, Dipp = 2,6‐*^i^* Pr_2_C_6_H_3_) with B_2_F_4_ (generated in situ), CpBeBeCp, or borazine led to the formation of Sn[B]_2_(BF_2_)_2_ (**2**), Sn[B]_2_(BeCp)_2_ (**3**), or Sn[B]_2_(Bz)(H) (**4**; Bz = B_3_N_3_H_5_), respectively (Scheme 1).^[^ ^32^, ^36^, ^44^ ^]^ Crystals of all three novel complexes of suitable quality for single‐crystal X‐ray diffraction (SC XRD) measurements could be obtained, allowing for unambiguous determination of their molecular structures in the solid state (Figure 1).

**Scheme 1:**
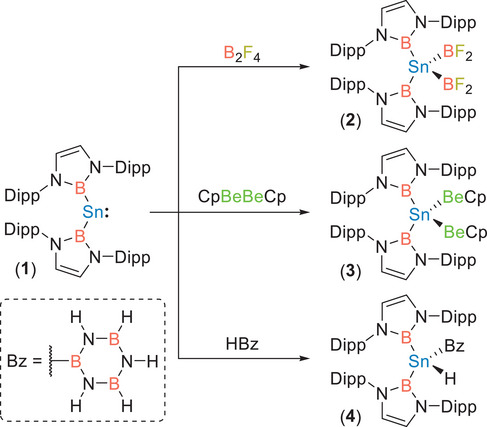
Synthesis of complexes **2**, **3**, and **4** from stannylene **1**.

**Figure 1:**
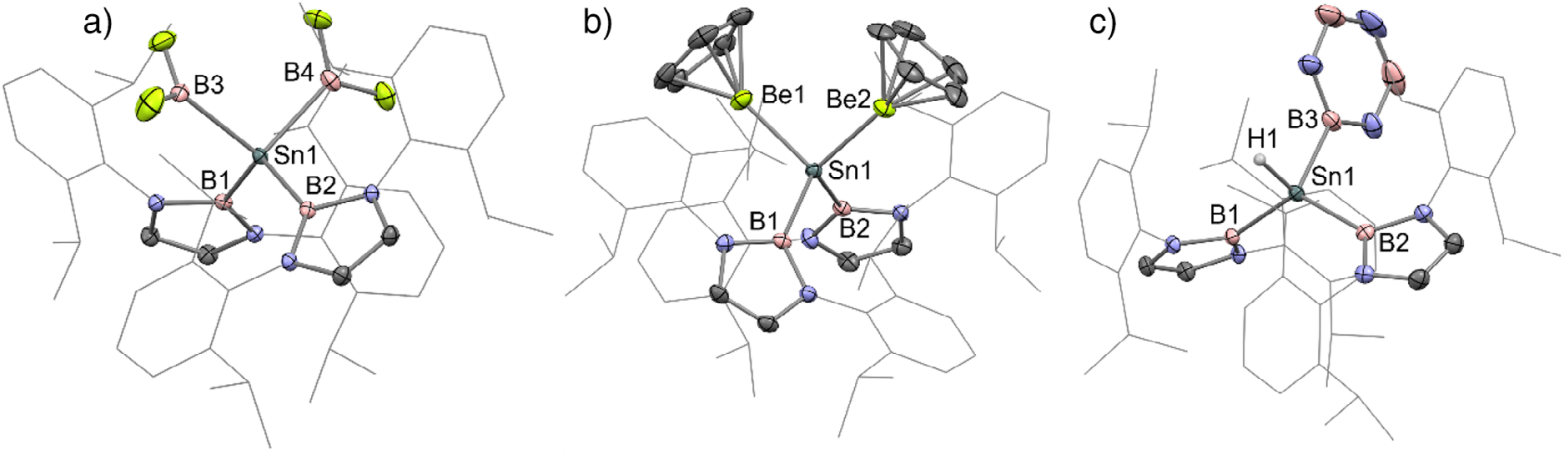
Molecular structures of complexes **2** a), **3** b), and **4** c), as determined by X‐ray crystallography. Selected hydrogen atoms omitted and selected groups represented in the wireframe format for clarity; thermal ellipsoids at the 50% probability level. Selected bond lengths (Å) and angles (°) for **2**: Sn1─B1 2.255(2), Sn1─B2 2.267(2), Sn1─B3 2.277(2), Sn1─B4 2.280(2), B3···B4 3.516(3), B1─Sn1─B2 124.69(8), B1─Sn1─B2 101.00(9); for **3**: Sn1─B1 2.289(2), Sn1─B2 2.286(2), Sn1─Be1 2.389(3), Sn1–Be2 2.388(3), Be1···Be2 3.577(6), B1─Sn1─B2 110.61(9), Be1─Sn1─Be2 96.97(12); for **4**: Sn1–B1 2.255(2), Sn1–B2 2.260(2), Sn1–B3 2.267(2), B1–Sn1–B2 128.37(8).

Complex **2** is a tetra(boryl) species with two diazaborolyl and two BF_2_ groups bonded to tin(IV) (Figure 1a). This complex is generated by the formal oxidative addition of the B─B bond of tetrafluorodiborane(4) to tin(II).^[^ ^44^, ^45^, ^46^, ^47^ ^]^ Despite there being considerable precedent for the activation of the B─B linkage by transition metal complexes, as well as by a base‐stabilised silylene,^[^ ^48^ ^]^ to our knowledge, this is the first example of the addition of this bond at a single main group metal centre.^[^ ^26^ ^]^ As such, **2** is of relevance to main group‐mediated borylation reactions, which are attracting increasing interest. Stable metal complexes of the difluoroboryl ligand remain extremely rare, although platinum and iridium systems have been structurally characterised.^[^ ^44^, ^46^, ^47^ ^]^ As an aside, the reaction of **1** with B_2_Cat_2_ was also examined; NMR spectroscopy indicates that this reaction is slow (80 °C, 72 h), likely due to steric congestion at Sn, but also leads to formal oxidative addition of the B─B bond, forming Sn[B]_2_(BCat)_2_ (see Supporting Information). In all cases, however, crystals of this compound were too poorly diffracting for satisfactory refinement of SC XRD data.

The geometry at Sn in complex **2** is distorted tetrahedral, with a wide B1–Sn1–B2 angle (124.69(8)°) and narrow B3–Sn1–B4 angle (101.00(9)°), apparently reflecting the different steric profiles of the different boryl ligands. The Sn1–B1 and Sn1–B2 distances (2.255(2) and 2.267(2) Å, respectively) are statistically indistinguishable from one another by the 3σ rule and are slightly shorter than the Sn1–B3 and Sn1–B4 distances (2.277(3) and 2.280(2) Å, respectively). These Sn–B distances are comparable to those measured for **1** (2.285(8) and 2.294(8) Å), despite the difference in formal oxidation state at Sn between these two complexes.^[^ ^36^, ^37^ ^]^ The B3···B4 distance is 3.516(3) Å—more than double the distance expected for a B─B single bond (1.7 Å)—indicating that the B─B bond of B_2_F_4_ has been fully cleaved.^[^ ^49^ ^]^

Similarly to **2**, **4** is also formally a tin(IV) complex (Figure 1c). Complex **4** is formed by the oxidative addition of a borazine B─H bond at tin(II)—to our knowledge, the first example of this reaction at any metal from across the Periodic Table—furnishing the tin centre with hydride and η^1^‐borazinyl ligands.^[^ ^37^, ^40^ ^]^ A very limited number of transition metal complexes of borazine derivatives are known.^[^ ^41^, ^42^, ^43^, ^50^, ^51^, ^52^ ^]^ For example, the complexes *trans*‐[M(B_3_N_3_Br_2_H_3_)(Br)(PCy_3_)_2_] (M = Pd, Pt) were prepared by oxidative addition of a B─Br bond of *B*‐tribromoborazine to [M(PCy_3_)_2_].^[^ ^41^ ^]^ Similarly to **4**, these transition metal complexes feature the borazinyl ligand bound to the respective metal centre in an η^1^‐manner through a single boron atom.

As with complex **2**, **4** features a highly distorted tetrahedral geometry, with a very wide B1–Sn1–B2 angle of 128.37(8)°. The three Sn–B distances are similar: Sn–B1, 2.255(2); Sn–B2, 2.260(2); Sn–B3, 2.267(2) Å. The electron density associated with the hydride ligand in this complex could be straightforwardly located in experimental SC XRD data. Moreover, multinuclear NMR data measured for **4** (*vide infra*) are consistent with the connectivity determined using SC XRD (i.e., the borazinyl ligand coordinates to Sn through the B atom, rather than N).

In contrast to **2** and **4**, complex **3** is formally a tin(0) complex, featuring two boryl and two beryllyl ligands (Figure 1b).^[^ ^7^ ^]^ Indeed, on the basis of the Pauling electronegativities of tin (1.96) and beryllium (1.57) the formation of **3** involves the “reductive” addition of a Be─Be bond at tin(II).^[^ ^6^, ^31^ ^]^ Although we have previously described the addition of Be─Be bonds to 3*d*‐metal centres, this is the first example of this bond addition at a main group metal.^[^ ^31^, ^35^ ^]^ Furthermore, very few beryllium‐metal bonding combinations are known, and complex **3** represents the first complex for which a Sn─Be bond has been structurally authenticated.^[^ ^12^, ^13^ ^]^

At 2.388(3) and 2.389(3) Å, the Sn1─Be1 and Sn1─Be2 distances in **3** are slightly shorter than the sum of the covalent radii calculated for these elements (2.42 Å).^[^ ^49^ ^]^ The Be1···Be2 distance in **3** is 3.577(6) Å, which signifies the absence of a covalent Be–Be interaction; such bonds are typically around 2.1 Å in length.^[^ ^32^, ^33^, ^34^ ^]^ The Sn1─B1 and Sn1─B2 distances of **3** (2.286(2) and 2.289(2) Å, respectively) are slightly longer than those in **2** and **4**, reflecting the more electron‐rich nature of the tin centre in **3**. Nonetheless, they are statistically indistinguishable from those in **1** (2.285(8) and 2.294(8) Å), despite the differences in the formal oxidation state of tin.^[^ ^37^ ^]^ The geometry at Sn in **3** is much closer to tetrahedral than that in **2** and **4**; the B1─Sn1─B2 and Be1─Sn1─Be2 angles are 110.61(9) and 96.97(12)°, respectively. Because the difluoroboryl, beryllyl, and hydride/borazinyl ligands in **2**–**4** do not have dramatically different steric profiles from one another, it could be suggested that the wide B1─Sn1─B2 angles in **2** and **4** are due to electronic factors, rather than the steric clash of the two bulky [B] ligands (*vide infra*).

Complexes **2**–**4** have also been characterised by multinuclear NMR spectroscopy. The ^1^H NMR spectrum of **4** is consistent with the binding of the borazinyl ligand to Sn through a boron atom (rather than nitrogen); resonances corresponding to one B─H hydride environment and two N─H proton environments integrate in a 2:3 ratio. ^117/119^Sn satellites are observed for the tin‐bound hydride resonance (1.67 ppm; ^1^ *J*_119SnH_ = 1175 Hz, ^1^ *J*_117SnH_ = 1122 Hz). Similarly, Sn coupling is observed in the ^9^Be NMR spectrum of **3**, which features one resonance at −24.5 ppm with a single set of unresolved ^117/119^Sn satellites (^1^ *J*_SnBe_ = 307 Hz).^[^ ^53^, ^54^ ^]^ Due to the coupling of Sn to several chemically inequivalent quadrupolar atoms, ^119^Sn resonances could not be observed for **2**–**4**.

With the synthesis of complexes **2**–**4** accomplished, we set out to examine the differing effects of the formally oxidative and reductive addition events upon the electronic structure at tin. Accordingly, we applied quantum chemical calculations (ωB97X‐D4 def2‐TZVP) to probe complexes **2**–**4**. For the purposes of comparison, calculations were also performed on tin(II) precursor **1**, tin(IV) di(hydride) complex Sn[B]_2_(H)_2_ (**5**), which is formed by the oxidative addition of the H─H bond of dihydrogen at **1**, and tin(IV) dichloride complex Sn[B]_2_(Cl)_2_ (**6**).^[^ ^36^, ^37^, ^55^ ^]^ In complexes **2**–**4**, tin engages in multicentre bonding with the four atoms to which it is covalently bonded. For example, the HOMO–12 of **2** involves five‐centre SnB_4_ bonding (Figure S23); the HOMO–3 of **3** represents a SnB_2_Be_2_ interaction (Figure S26); and the HOMO–12 of **4** corresponds to a four‐centre bond between tin, the hydride, and the two boron atoms of the [B] ligands (Figure S30). This adds credence to the hypothesis that the geometries of **2** – **4** are influenced by electronic factors and facilitate maximal Sn–E (E = B, Be, H) orbital overlap in these multicentre interactions. However, the differing radii of the Sn^II^ and Sn^0^ centres in **2**, **3**, and **4**, respectively, may also influence the observed geometries in these complexes.

Natural Bond Orbital (NBO) calculations were employed to further probe the nature of the Sn–E bonding in **2**–**4**. In the case of bis(BF_2_) complex **2**, NBO analysis returns four covalent Sn─B interactions, which are each composed of approximately 45:55 Sn:B character. This is mirrored by the compositions calculated for the three Sn─B bonds of (boryl)hydride **4**, whilst the parentage of the Sn–H interaction is 40:60 Sn:H. In the case of bis(beryllyl) system **3**, the contributions to the two Sn–B bonds are approximately equivalent to those calculated for **2** and **4**, and the Sn─Be bonds are of 71:29 Sn:Be character. In all complexes, the Sn contributions to Sn–E bonds are calculated to comprise 20–30% 5*s*‐ and 70–80% 5p‐orbital character.

The charge distribution in **1**–**6** was evaluated by Natural Population Analysis (NPA) and Quantum Theory of Atoms in Molecules (QTAIM) calculations, the results of which are displayed in Table 1. The Natural Electron Configurations (NECs) of the tin centres in these complexes are also presented. Across complexes **2**–**6**, the average NPA (+0.45 to + 0.63) and Bader (+1.22 to + 1.33) charges at the boron atoms of the [B] ligands change very little. Notably, however, the charge of the tin centre varies significantly from one complex to another; the lowest charge is calculated for the tin centre of bis(beryllyl) complex **3** (NPA, −0.65; Bader, −0.77) and the most positive for the tin centre of bis(chloro) complex **6** (NPA, +1.30; Bader, +1.36). As already discussed, based on electronegativity arguments, the addition of the Be─Be bond to Sn(II) is formally a reductive process. By the same reasoning, the addition of a Cl─Cl bond to the Sn centre of **1** is a clear‐cut example of an oxidative reaction. By inspection of the charge distribution data in Table 1, however, it is apparent that not all of these bond addition reactions conform to this binary classification system. Indeed, it could be asserted that the natures of B─B, B─H, and H─H bond addition processes fall on a spectrum between the two “oxidative” and “reductive” extremes (Figure 2).

**Table 1:** Natural Electron Configuration of the Sn atom and charge distributions in complexes **1**–**5**.

		Charge at Sn		Av. charge at B_[B]_		Av. charge at E	
Complex	Natural Electron Configuration of Sn	NPA	Bader	NPA	Bader	NPA	Bader
**Sn[B]_2_ (1)**	[core]5s(1.65)5p(1.56)	+0.76	+0.59	+0.28	+1.18	–	–
**Sn[B]_2_(BF_2_)_2_ (2)**	[core]5s(1.14)5p(2.56)	+0.29	+0.83	+0.58	+1.28	+1.00	+1.43
**Sn[B]_2_(BeCp)_2_ (3)**	[core]5s(1.33)5p(3.28)	−0.65	−0.77	+0.63	+1.22	+1.27	+1.48
**Sn[B]_2_(Bz)(H) (4)**	[core]5s(1.06)5p(2.37)	+0.55	+0.91	+0.58	+1.26	H, −0.20 B, +0.67	H, −0.66 B, +1.38
**Sn[B]_2_(H)_2_ (5)**	[core]5s(1.11)5p(2.26)	+0.62	+1.08	+0.52	+1.27	−0.20	−0.38
**Sn[B]_2_(Cl)_2_ (6)**	[core]5s(1.09)5p(1.58)	+1.30	+1.36	+0.45	+1.33	−0.51	−0.63

B_[B]_ denotes the boron atom of the [B] ligand; E denotes the boron atom of the BF_2_ group or borazinyl moiety, Be, H, or Cl. The charge of E in complex **4** is not an average—the values for both B and H are displayed.

**Figure 2:**
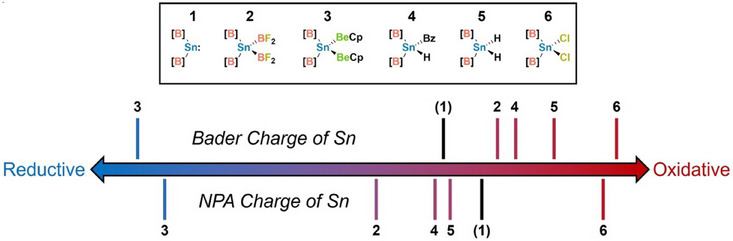
Illustration of an 'oxidative' to ’reductive’ reaction spectrum for the addition of E–E’ bonds to the Sn^II^ centre of complex **1**. The relative positions of markers are based on the calculated charge at the Sn centre within the denoted complex.

As would be intuitively predicted, the Sn centre of bis(boryl) complex **2** (NPA, +0.29) is calculated to be less electron‐rich than that within bis(beryllyl) complex **3**. Moreover, calculations also indicate that the tin centres of (boryl)hydride complex **4** (NPA, +0.55) and of di(hydride) complex **5** (NPA, +0.62) are more oxidised than that of **2**. Interestingly, the NPA charges of the Sn centres in complexes **2**, **4**, and **5** are actually lower than that calculated for precursor **1** (+0.76), which would indicate the partial reduction of the Sn^II^ centre upon addition of B─B, B─H, and H─H bonds. However, although general trends remain the same, Bader charges calculated for the Sn centres of complexes **2** (+0.83), **4** (+0.91), and **5** (+1.08) are more positive than that for complex **1** (+0.59). Irrespective, Figure 2 illustrates that a nuanced description is required in order to articulate the true nature of several of these bond addition processes.

In conclusion, we have investigated formally oxidative (B─B, B─H, H─H, Cl─Cl) and reductive bond addition (Be─Be) reactions at a bis(boryl)stannylene. The Be─Be and B─B bond additions are the first experimentally reported examples of such reactions at a main group metal centre. We also demonstrate the first example of borazine B─H bond addition to any element centre. Density functional theory calculations indicate that the nature of the addition process can be considered to lie on a spectrum from unambiguously “oxidative” (Cl─Cl) to “reductive” (Be─Be), with the other bond addition reactions (B─B, B─H, H─H) sitting between these two extremes.

## Conflict of Interests

The authors declare no conflict of interest.

## Supporting information



Supporting Information

## Data Availability

The data that support the findings of this study are available in the supplementary material of this article.
