# Supplementary material for: Breaking Bonds at Tin(II): Reductive or Oxidative Addition?
Source: Angew Chem Int Ed Engl. 2025 Jun 12;64(32):e202503050. doi: 10.1002/anie.202503050 (PMC12322635; doi:10.1002/anie.202503050)
Supplement: Supplementary file 1 — Supporting Information [file ANIE-64-e202503050-s001.docx]

**Supporting Information for**

**Breaking Bonds at Tin(II): Reductive or Oxidative Addition?**

Maximilian Dietz,*^,a,†^ Josef T. Boronski,*^,b,†^ Amelia M. Swarbrook,^a^ Simon Aldridge*^,a^

^a^ *Inorganic Chemistry Laboratory, Department of Chemistry, University of Oxford, South Parks Road, Oxford, OX1 3QR (UK).*

^b^ *Department of Chemistry, Molecular Sciences Research Hub, Imperial College London, 82 Wood Lane, White City, London, W12 0BZ (UK).*

^†^ *these authors contributed equally to this work.*

**Contents**

[Methods and materials 2](#_Toc196301399)

[Synthetic procedures 3](#_Toc196301400)

[NMR spectra of isolated compounds 7](#_Toc196301401)

[X-ray crystallographic data 24](#_Toc196301402)

[Computational details 29](#_Toc196301403)

[Coordinates of Optimized Structures 35](#_Toc196301404)

[References 44](#_Toc196301405)

# Methods and materials

All manipulations were performed either under an atmosphere of dry argon or dinitrogen using standard Schlenk line or glovebox techniques. Solvents were purified and dried using an Mbraun SPS-800, degassed before use and stored over 3 Å molecular sieves. Deuterated solvents were dried over 3 Å molecular sieves, degassed by three freeze-pump-thaw cycles and stored under inert atmosphere in a Teflon valve ampoule. NMR samples were prepared under inert atmosphere in 5 mm Wilmad 507-PP tubes fitted with J. Young Teflon valves. Liquid-phase NMR spectra were acquired on a Bruker Avance III 500 (^1^H: 499.9 MHz, ^9^Be: 70.2 Hz, ^11^B: 160.4 MHz, ^14^N: 36.1 MHz, ^19^F: 470.4 MHz, ^13^C: 125.7 MHz, ^119^Sn: 186.4 MHz) or a Bruker Avance III 700 (^1^H: 699.9 MHz) spectrometer. Chemical shifts (*δ*) are reported in ppm and internally referenced to the carbon nuclei (^13^C{^1^H}) or residual protons (^1^H) of the solvent. Heteronuclei NMR spectra are referenced to external standards (^9^Be: BeSO_4_, ^11^B: BF_3_$\cdot$OEt_2_, ^14^N: MeNO_2_, ^19^F: CFCl_3_, ^119^Sn: Me_4_Sn). Resonances are given as singlet (s), doublet (d), triplet (t), septet (sept), multiplet (m) or broad (br).

Solvents and reagents were purchased from Sigma-Aldrich, Fisher Scientific, TCI or Alfa Aesar. [B]_2_Sn (**1**, [B] = B(NDippCH)_2_),^[1]^ B_2_Br_4_(SMe_2_)_2_,^[2]^ Be_2_Cp_2_,^[3]^ and borazine^[4]^ were synthesized using literature procedures.

**Health warning:** beryllium and its compounds are extremely toxic and can cause irreversible health effects through inhalation or skin contact. The work with beryllium-containing materials described herein was carried out by trained operator(s), with strict adherence to local and national rules/regulations. The toxicity of the beryllium-containing compounds precludes their characterization by elemental analysis.

# Synthetic procedures

**Synthesis of [B]_2_Sn(BF_2_)_2_** (**2**)

**1** (50.0 mg, 56.0 μmol) was placed in one side of a H tube, while the other side was loaded with a solid mixture of B_2_Br_4_(SMe_2_)_2_ (78.2 mg, 168 mmol) and SbF_3_ (39.9 mg, 223 mmol). Toluene (2 mL) was added to each side of the H tube, which was subsequently frozen in liquid nitrogen, evacuated and closed. After warming up to room temperature, the side of the H tube containing the diborane precursor was ultrasonicated at room temperature for 20 min, while the other side was cooled to –78 °C. The *in situ* generated solution of B_2_F_4_ was subsequently vacuum transferred onto the stannylene solution under static vacuum. The reaction mixture was warmed up to room temperature, which led to a colour change to pale yellow. All volatiles were removed *in vacuo*, the beige residue extracted with pentane (5 mL) and quick evaporation of the solvent led to the formation of crystals suitable for X-ray diffraction analysis. The crystals were washed with pentane (0.5 mL) and dried at atmospheric pressure, which gave **2** as beige crystals (21.1 mg, 21.3 μmol, 38%). *Note: The product is sensitive to reduced pressure both in solution and in the solid state, which precluded removal of remaining pentane for NMR analysis. Different decomposition products could be observed with [B]_2_SnF_2_ and [B]_2_Sn(BF_2_)(F) presumably being the major species.*

**^1^H NMR** (499.9 MHz, C_6_D_6_, 298 K): *δ* = 7.19 (t, ^3^*J*_H–H_ = 7.7Hz, 4H, *p*‑Dipp*H*), 7.09 (d, ^3^*J*_H–H_ = 7.7 Hz, 8H, *m*‑Dipp*H*), 6.16 (s + satellites, ^4^*J*_119/117Sn–H_ = 9.4 Hz, 4H, NC*H*), 3.02 (sept, ^3^*J*_H–H_ = 6.8 Hz, 8H, *i*Pr‑C*H*), 1.15 (d, ^3^*J*_H–H_ = 6.8 Hz, 24H, *i*Pr‑C*H*_3_), 1.09 (d, ^3^*J*_H–H_ = 6.8 Hz, 24H, *i*Pr‑C*H*_3_) ppm.

**^13^C{^1^H} NMR** (125.7 MHz, C_6_D_6_, 298 K): *δ* = 146.0 (*o*‑Dipp*C*), 140.2 (*i*‑Dipp*C*), 128.4 (*p*‑Dipp*C*), 124.3 (*m*‑Dipp*C*), 124.0 (s + satellites, ^3^*J*_119/117Sn–C_ = 31.6 Hz, N*C*H), 28.8 (*i*Pr‑*C*H), 26.7 (*i*Pr‑*C*H_3_), 23.1 (*i*Pr‑*C*H_3_) ppm.

**^11^B NMR** (160.4 MHz, C_6_D_6_, 298 K): *δ* = 27.2 (br s) ppm.

**^19^F{^1^H} NMR** (470.4 MHz, C_6_D_6_, 298 K): *δ* = –14.2 (br s) ppm.

**^119^Sn{^1^H} NMR** (186.4 MHz, C_6_D_6_, 298 K): *δ* = n.d.

**Synthesis of [B]_2_Sn(BCat)_2_**

**1** (50.0 mg, 56.0 μmol) and B_2_Cat_2_ (14.7 mg, 46.7 μmol) were combined in benzene (5 mL) and the solution was heated 80 °C for 3 d, whereupon the colour changed to light brown. All volatiles were removed *in vacuo* and the residue was extracted with pentane (2 mL). Slow evaporation of the solution led to the precipitation of a microcrystalline solid, which was washed with pentane (0.5 mL) and dried *in vacuo*, yielding [B]_2_Sn(BCat)_2_ as a beige solid (52.8 mg,51.7 μmol, 77%).

**^1^H NMR** (499.9 MHz, C_6_D_6_, 298 K): *δ* = 6.91 (d, ^3^*J*_H–H_ = 7.6 Hz, 8H, *m*‑Dipp*H*), 6.88–6.79 (m, 12H, *p*‑Dipp*H* + Cat*H*), 6.19 (s + satellites, ^4^*J*_119/117Sn–H_ = 8.8 Hz, 4H, NC*H*), 3.15 (br s, 8H, *i*Pr‑C*H*), 1.14 (d, ^3^*J*_H–H_ = 6.8 Hz, 24H, *i*Pr‑C*H*_3_), 1.10 (d, ^3^*J*_H–H_ = 6.8 Hz, 24H, *i*Pr‑C*H*_3_) ppm.

**^13^C{^1^H} NMR** (125.7 MHz, C_6_D_6_, 298 K): *δ* = 149.1 (s + satellites, ^3^*J*_119/117Sn–C_ = 17.9 Hz, Cat*C*_q_), 146.0 (s + satellites, ^3^*J*_119/117Sn–C_ = 21.5 Hz, *o*‑Dipp*C*), 140.4 (*i*‑Dipp*C*), 127.5 (*p*‑Dipp*C*), 123.8 (*m*‑Dipp*C*), 123.8 (s + satellites, ^3^*J*_119/117Sn–C_ = 31.5 Hz, N*C*H), 121.7 (Cat*C*H), 112.6 (Cat*C*H), 28.7 (*i*Pr‑*C*H), 26.9 (*i*Pr‑*C*H_3_), 23.1 (*i*Pr‑*C*H_3_) ppm.

**^11^B NMR** (160.4 MHz, C_6_D_6_, 298 K): *δ* = 38.8 (br s, *B*Cat), 27.9 (br s, *B*(NDippCH)_2_) ppm.

**^119^Sn{^1^H} NMR** (186.4 MHz, C_6_D_6_, 298 K): *δ* = n.d.

**Synthesis of [B]_2_Sn(BeCp)_2_ (3)**

A J Young‘s NMR tube was charged with a solid mixture of diberyllocene (1.0 mg, 6.72 μmol) and **1** (6.0 mg, 6.72 μmol). Benzene-d_6_ was added (0.4 mL) and the yellow solution turned colourless within 10 s. After 1 h of ultrasonic agitation, all volatiles were removed *in vacuo*, yielding a colourless solid. Soluble material was extracted with hexane (1 mL) and the solution was filtered. Slow evaporation of the solvent over the course of 16 h led to the formation of a crop of colourless crystals of **3**, which were dried *in vacuo* (5.8 mg, 5.57 μmol, 82%). Single crystals of **3** suitable for X-ray diffraction experiments were obtained by slow evaporation of a benzene/hexane (1:1) solution.

**^1^H NMR** (499.9 MHz, C_6_D_6_, 298 K): *δ* = 7.23 (d, 8H, *m*‑Dipp*H*), 7.12 (t, ^3^*J*_H–H_ = 7.7 Hz, 4H, *p*‑Dipp*H*), 5.99 (s, 4H, NC*H*), 5.46 (s, 10H, C_5_*H*_5_), 3.62 (sept, 2H, *i*Pr‑C*H*), 3.12 (sept, 2H, *i*Pr‑C*H*), 2.80 (sept, 2H, *i*Pr‑C*H*), 2.71 (sept, 2H, *i*Pr‑C*H*), 1.38 (s, 6H, *i*Pr‑C*H*_3_), 1.31–1.11 (m, 36H, *i*Pr‑C*H*_3_), 0.68 (s, *i*Pr‑C*H*_3_) ppm.

**^13^C{^1^H} NMR** (125.7 MHz, C_6_D_6_, 298 K): *δ* = 145.9 (*o*‑Dipp*C*), 140.3 (*i*‑Dipp*C*), 127.8 (*p*‑Dipp*C*), 124.2 (*m*‑Dipp*C*), 123.3 (s, N*C*H), 104.3 (*C*_5_Me_5_), 28.1 (*i*Pr‑*C*H), 23.5 (*i*Pr‑*C*H_3_) ppm.

**^9^Be NMR** (70.2 MHz, C_6_D_6_, 298 K): *δ* = –24.5 (s + satellites, ^1^*J*_119/117Sn–Be_ = 306 Hz, *ω*_1/2_ = 51.3 Hz) ppm.

**^11^B NMR** (160.4 MHz, C_6_D_6_, 298 K): *δ* = 29.7 (br s) ppm.

**^119^Sn{^1^H} NMR** (186.4 MHz, C_6_D_6_, 298 K): *δ* = n.d.

**Synthesis of borazine**

Borazine was synthesized according to a reported literature procedure. The data match those reported previously.^[3]^

**^1^H NMR** (699.9 MHz, C_6_D_6_, 298 K): *δ* = 5.08 (t, ^1^*J*_14N–H_ = 54 Hz, 3H, N*H*), 4.46 (q, ^1^*J*_11B–H_ = 139 Hz, 3H, B*H*).

**^11^B NMR** (160.4 MHz, C_6_D_6_, 298 K): *δ* = 30.5 (d, ^1^*J*_11B–H_ = 139 Hz).

**^14^N{^1^H} NMR** (36.1 MHz, C_6_D_6_, 298 K): *δ* = 71.5 (s).

**Synthesis of [B]_2_Sn(Bz)(H) (4)**

A solution of **1** (20.0 mg, 22.4 μmol) in benzene was treated with a stock solution of borazine in benzene (181 μL, 22.4 μmol, *c* = 124 μmol mL^–1^), whereupon the reaction mixture became colourless. All volatiles were removed *in vacuo* and the residue was extracted with hexane (2 mL). Slow evaporation of this solution led to the formation of colourless crystals suitable for X-ray diffraction analysis, which were washed with hexane (0.5 mL) and dried at atmospheric pressure, yielding **4** as a colourless solid (18.0 mg, 18.5 μmol, 83%).

**^1^H NMR** (499.9 MHz, C_6_D_6_, 298 K): *δ* = 7.19 (t, ^3^*J*_H–H_ = 7.7Hz, 4H, *p*‑Dipp*H*), 7.09 (d,
^3^*J*_H–H_ = 7.7 Hz, 4H, *m*‑Dipp*H*), 7.04 (d, ^3^*J*_H–H_ = 7.7 Hz, 4H, *m*‑Dipp*H*), 6.20 (s + satellites, ^4^*J*_119/117Sn–H_ = 8.1 Hz, 4H, NC*H*), 4.92 (br s, 1H, N*H*), 4.68 (br s, 2H, N*H*), 4.22 (br s, 2H, B*H*), 3.29 (sept, ^3^*J*_H–H_ = 6.8 Hz, 4H, *i*Pr‑C*H*), 2.95 (sept, ^3^*J*_H–H_ = 6.8 Hz, 4H, *i*Pr‑C*H*), 1.67 (s + satellites, ^1^*J*_119Sn–H_ = 1175 Hz, ^1^*J*_117Sn–H_ = 1122 Hz, 1H, Sn*H*), 1.20 (d, ^3^*J*_H–H_ = 6.8 Hz, 12H, *i*Pr‑C*H*_3_), 1.09 (d, ^3^*J*_H–H_ = 6.8 Hz, 24H, *i*Pr‑C*H*_3_), 1.02 (d, ^3^*J*_H–H_ = 6.8 Hz, 12H, *i*Pr‑C*H*_3_) ppm.

**^13^C{^1^H} NMR** (125.7 MHz, C_6_D_6_, 298 K): *δ* = 146.2 (*o*‑Dipp*C*), 145.6 (*o*‑Dipp*C*), 141.2 (*i*‑Dipp*C*), 127.8 (*p*‑Dipp*C*), 124.5 (*m*‑Dipp*C*), 123.6 (*m*‑Dipp*C*), 123.1 (s + satellites, ^3^*J*_119/117Sn–C_ = 30 Hz, N*C*H), 28.9 (*i*Pr‑*C*H), 28.7 (*i*Pr‑*C*H), 26.2 (*i*Pr‑*C*H_3_), 25.8 (*i*Pr‑*C*H_3_), 23.8 (*i*Pr‑*C*H_3_), 23.0 (*i*Pr‑*C*H_3_) ppm.

**^11^B NMR** (160.4 MHz, C_6_D_6_, 293 K): *δ* = 40.0 (v br s), 29.4 (br s) ppm.

**^119^Sn{^1^H} NMR** (186.4 MHz, C_6_D_6_, 293 K): *δ* = n.d.

# NMR spectra of isolated compounds


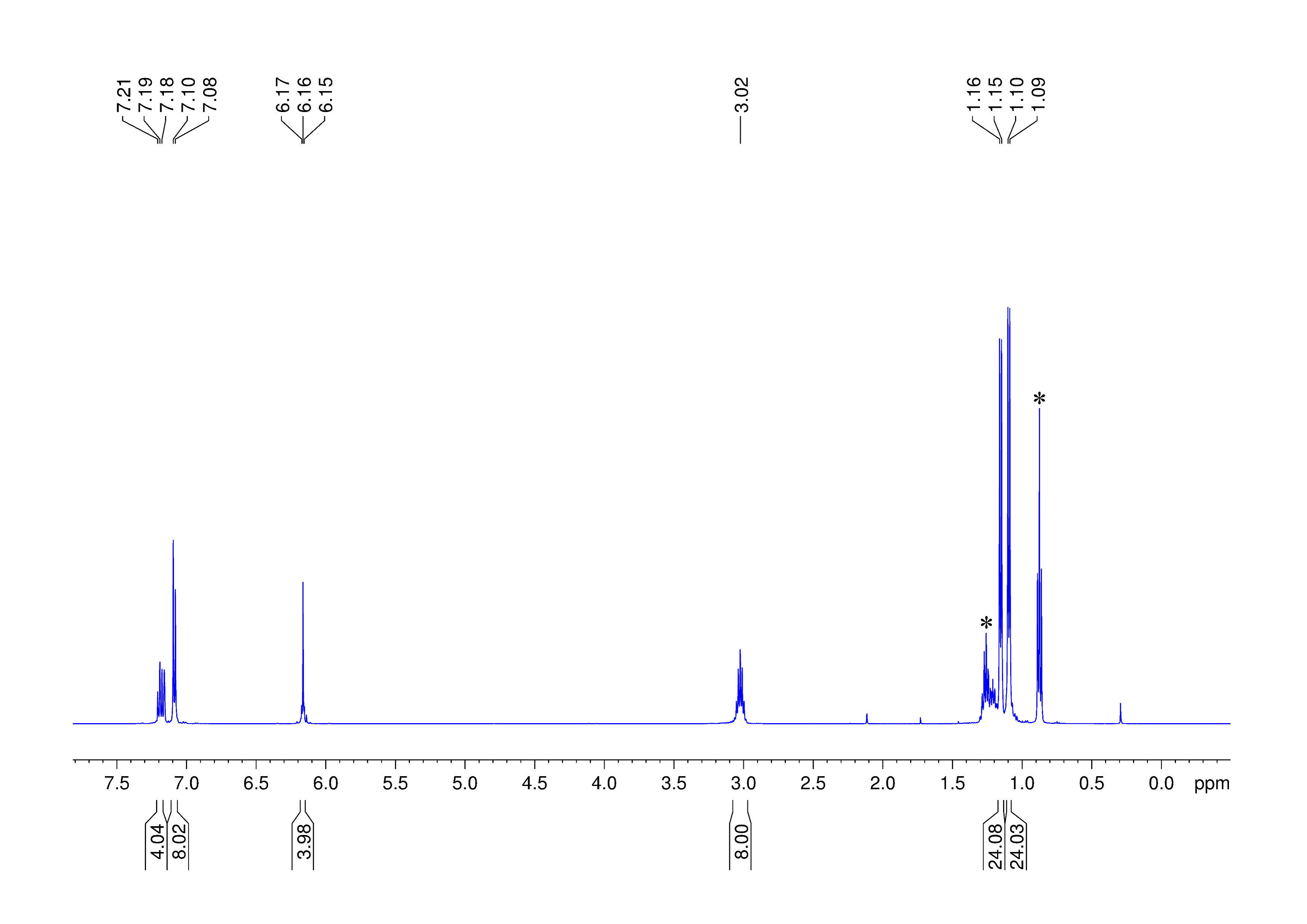


**Figure S1.** ^1^H NMR spectrum of **2** in C_6_D_6_ at 298 K (residual pentane marked with *).


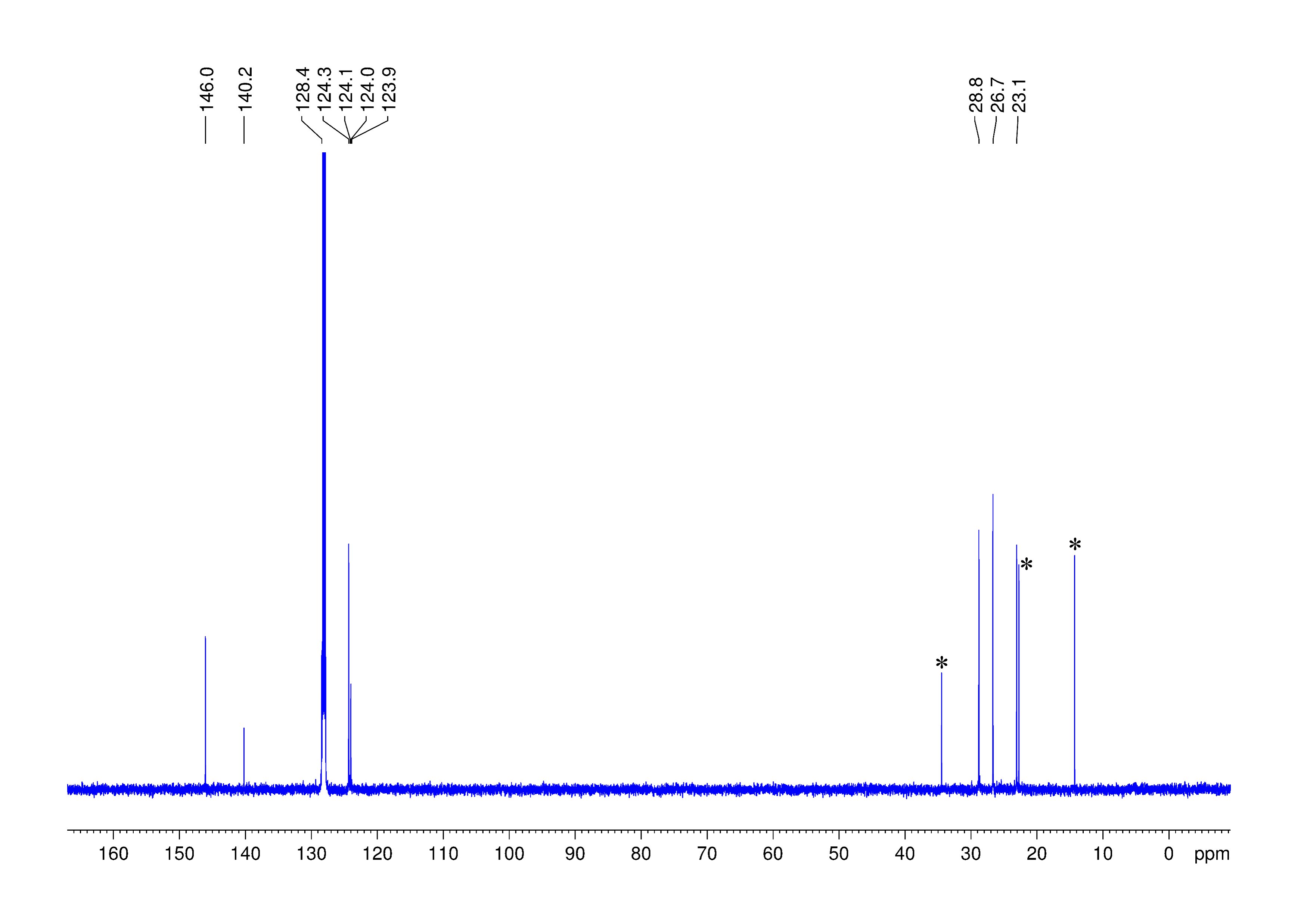


**Figure S2.** ^13^C{^1^H} NMR spectrum of **2** in C_6_D_6_ at 298 K (residual pentane marked with *).





**Figure S3.** ^11^B NMR spectrum of **2** in C_6_D_6_ at 298 K.





**Figure S4.** ^19^F{^1^H} NMR spectrum of **2** in C_6_D_6_ at 298 K.





**Figure S5.** ^1^H NMR spectrum of [B]_2_Sn(BCat)_2_ in C_6_D_6_ at 298 K.





**Figure S6.** ^13^C{^1^H} NMR spectrum of [B]_2_Sn(BCat)_2_ in C_6_D_6_ at 298 K.





**Figure S7.** ^11^B NMR spectrum of [B]_2_Sn(BCat)_2_ in C_6_D_6_ at 298 K.

**Figure S8.** ^1^H NMR spectrum of **3** in C_6_D_6_ at 298 K.

**Figure S9.** ^13^C{^1^H} NMR spectrum of **3** in C_6_D_6_ at 298 K.

**Figure S10.** ^9^Be NMR spectrum of **3** in C_6_D_6_ at 298 K.

**Figure S11.** ^11^B NMR spectrum of **3** in C_6_D_6_ at 298 K.





**Figure S12**. ^1^H NMR spectrum of borazine in C_6_D_6_ at 298 K.





**Figure S13.** ^11^B NMR spectrum of borazine in C_6_D_6_ at 298 K.





**Figure S14.** ^15^N{^1^H} NMR spectrum of borazine in C_6_D_6_ at 298 K.


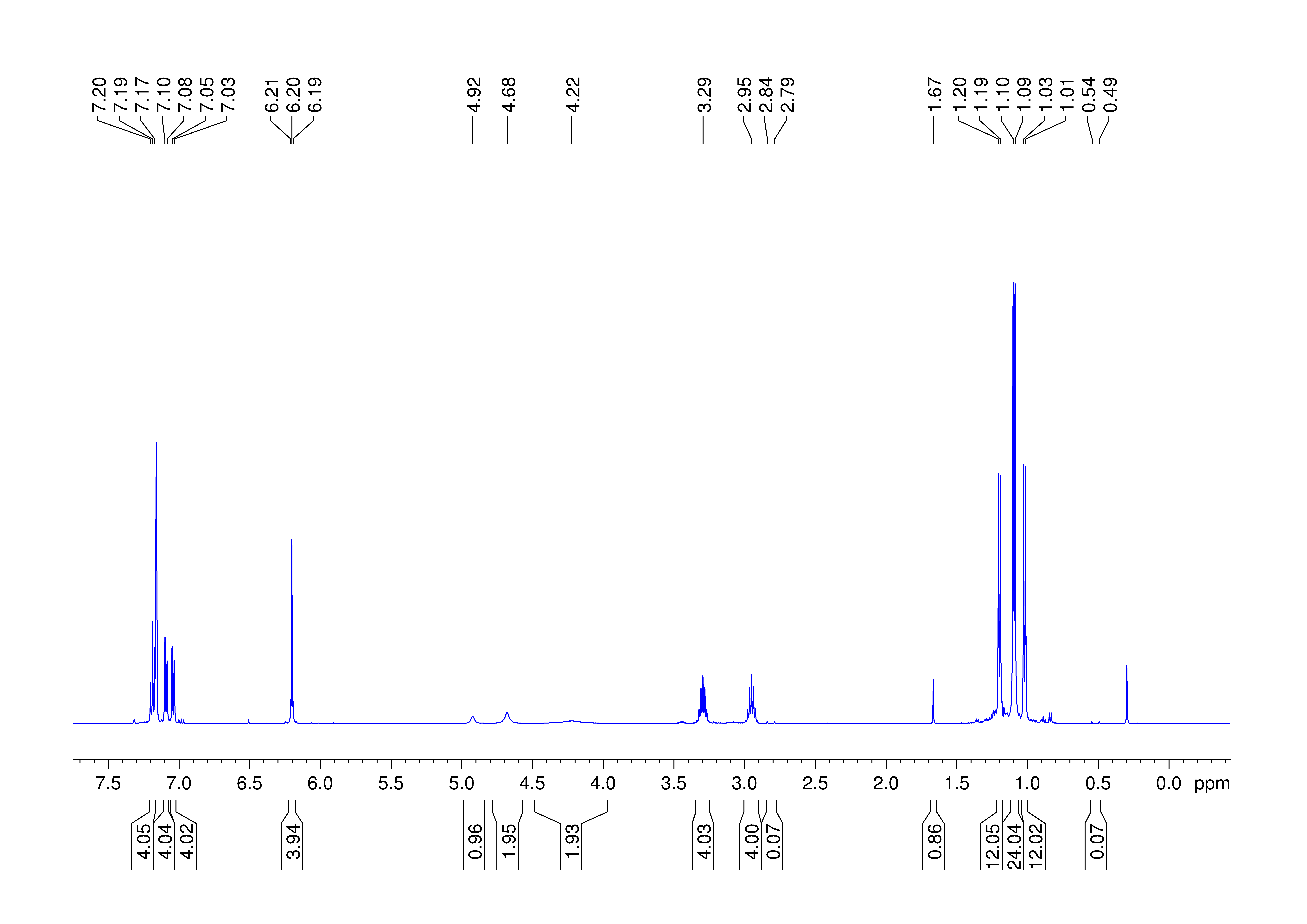


**Figure S15.** ^1^H NMR spectrum of **4** in C_6_D_6_ at 298 K.





**Figure S16.** ^13^C{^1^H} NMR spectrum of **4** in C_6_D_6_ at 298 K.

**
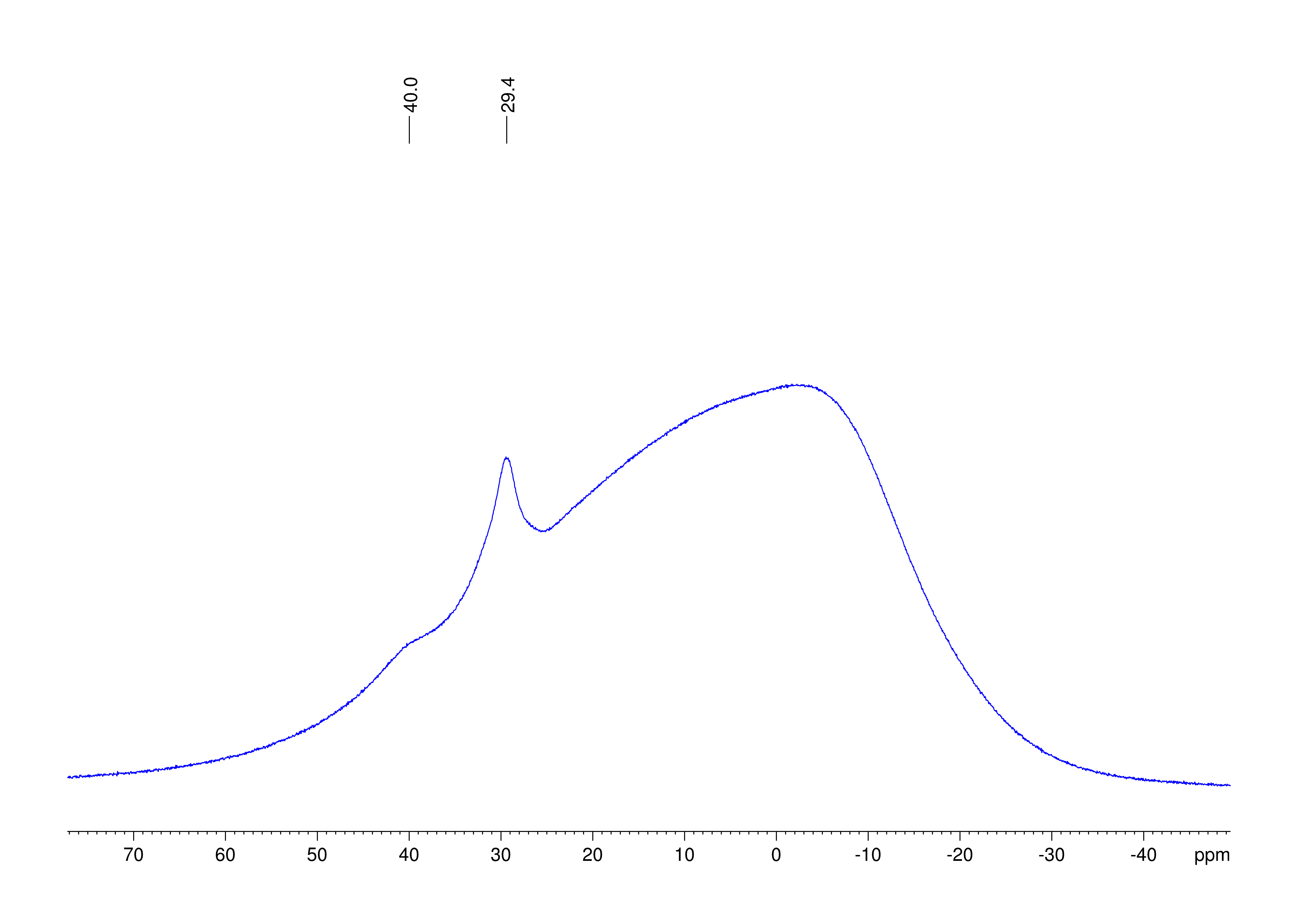
**

**Figure S17.** ^11^B NMR spectrum of **4** in C_6_D_6_ at 298 K.

# X-ray crystallographic data

Single-crystal X-ray diffraction data for compounds **2**, **3** and **4** were collected on either an Oxford Diffraction/Agilent SuperNova diffractometer equipped with a 135 mm Atlas CCD area detector or a Rigaku XtaLAB Synergy-DW VHF equipped with a PhotonJet-R dual wavelength rotating anode and HyPix-Arc 150° detector. Crystals were prepared and selected under Paratone-N or perfluorinated oil, mounted on MiTeGen MicroMount loops and quench-cooled using an Oxford Cryosystems open flow N_2_ cooling device.^[5]^ Data were collected at either 100 K or 150 K using monochromated Cu_Kα_ radiation (*λ* = 1.54184 Å) and processed using the CrysAlisPro package, including unit cell parameter refinement and inter-frame scaling (which was carried out using SCALE3 ABSPACK within CrysAlisPro). Equivalent reflections were merged and diffraction patterns processed with the CrysAlisPro suite.^[6]^ The structures were solved using the intrinsic phasing method,^[7]^ refined with SHELXL^[8-9]^ within the graphical interface ShelXle^[10]^ by using the *full-matrix-least-squares-on-F^2^* method and expanded using Fourier techniques. All non-hydrogen atoms were refined anisotropically. Hydrogen atoms were included in structure factor calculations. All hydrogen atoms were assigned to idealized geometric positions. Selected crystallographic data are summarised in **Table S1**.

Crystallographic data have been deposited with the Cambridge Crystallographic Data Centre as supplementary publication numbers CCDC-2418817 (**2**), 2418818 (**3**), 2418819 (**4**). These data can be obtained free of charge from The Cambridge Crystallographic Data Centre *via* www.ccdc.cam.ac.uk/data_request/cif.

**Table S1.** Crystal structure data and structure refinement parameters of compouds **2** – **4**.

| **Compound** | **2** | **3** | **4** |
| --- | --- | --- | --- |
| CCDC number | 2418817 | 2418818 | 2418819 |
| Empirical formula | C_52_H_72_B_4_F_4_N_4_Sn | C_68_H_88_B_2_Be_2_N_4_Sn | C_58_H_89_._25_B_5_N_7_Sn |
| Formula weight  [g mol^–1^] | 991.06 | 1119.75 | 1057.34 |
| Temperature [K] | 150(2) | 100(2) | 100(2) |
| Radiation *λ* [Å] | Cu_Kα_, 1.54184 | Cu_Kα_, 1.54184 | Cu_Kα_, 1.54184 |
| Crystal system | monoclinic | hexagonal | triclinic |
| Space group | *P*2_1_/*n* | *P*6_5_ | *P*‾1 |
| *Unit cell dimensions* |  |  |  |
| *a* [Å] | 13.5014(3) | 13.25330(10) | 12.04710(10) |
| *b* [Å] | 21.5523(4) | 13.25330(10) | 12.99270(10) |
| *c* [Å] | 18.7131(4) | 61.0426(3) | 20.8514(2) |
| *α* [°] | 90 | 90 | 76.5290(10) |
| *β* [°] | 106.868(3) | 90 | 74.3380(10) |
| *γ* [°] | 90 | 120 | 74.3860(10) |
| Volume [Å^3^] | 5211.0(2) | 9285.63(15) | 2981.05(5) |
| *Z* | 4 | 6 | 2 |
| Calculated density  [Mg m^–3^] | 1.263 | 1.201 | 1.178 |
| Absorption coefficient [mm^–1^] | 4.312 | 3.591 | 3.710 |
| *F*(000) | 2072 | 3552 | 1122 |
| Theta range for collection | 3.591 to 70.074° | 3.851 to 72.123° | 2.234 to 70.071° |
| Reflections collected | 25963 | 73950 | 64497 |
| Unique reflections | 9883 | 12062 | 11328 |
| Unique reflections with  [*I* > 2*σ*(*I*)] | 8481 | 11883 | 10604 |
| Minimum/maximum transmission | 0.432/1.000 | 0.707/1.000 | 0.345/1.000 |
| Refinement method | Full-matrix least-squares on *F*^2^ | Full-matrix least-squares on *F*^2^ | Full-matrix least-squares on *F*^2^ |
| Data / parameters / restraints | 9883 / 602 / 0 | 12062 / 710 / 1 | 11328 / 759 / 508 |
| *Goodness-of-fit* on *F*^2^ | 1.038 | 1.070 | 1.091 |
| Final *R* indices  [*I* > 2*σ*(*I*)] | *R_1_* = 0.0257, *wR_2_* = 0.0621 | *R_1_* = 0.0176, *wR_2_* = 0.0453 | *R_1_* = 0.0295, *wR_2_* = 0.0790 |
| *R* indices (all data) | *R_1_* = 0.0337, *wR_2_* = 0.0669 | *R_1_* = 0.0180, *wR_2_* = 0.0454 | *R_1_* = 0.0320, *wR_2_* = 0.0806 |
| Maximum/minimum residual  electron density (e·Å^–3^) | 0.381 / –0.301 | 0.240 / –0.211 | 0.941 / –0.468 |

-----------------------------------------------------------------------------------------------------------------

**Crystal data for 2:** C_52_H_72_B_4_F_4_N_4_Sn, *M*_r_ = 991.06, colourless block, 0.260×0.180×0.090 mm^3^, monoclinic space group *P*2_1_/*n*, *a* = 13.5014(3) Å, *b* = 21.5523(4) Å, *c* = 18.7131(4) Å, *β* = 106.868(3)°, *V* = 5211.0(2) Å^3^, *Z* = 4, *ρ_calcd_* = 1.263 g·cm^–3^, *μ* = 4.312 mm^–1^, *F*(000) = 2072, *T* = 150(2) K, *R_1_* = 0.0337, *wR_2_* = 0.0669, 9883 independent reflections [2*θ*≤140.148°] and 602 parameters.


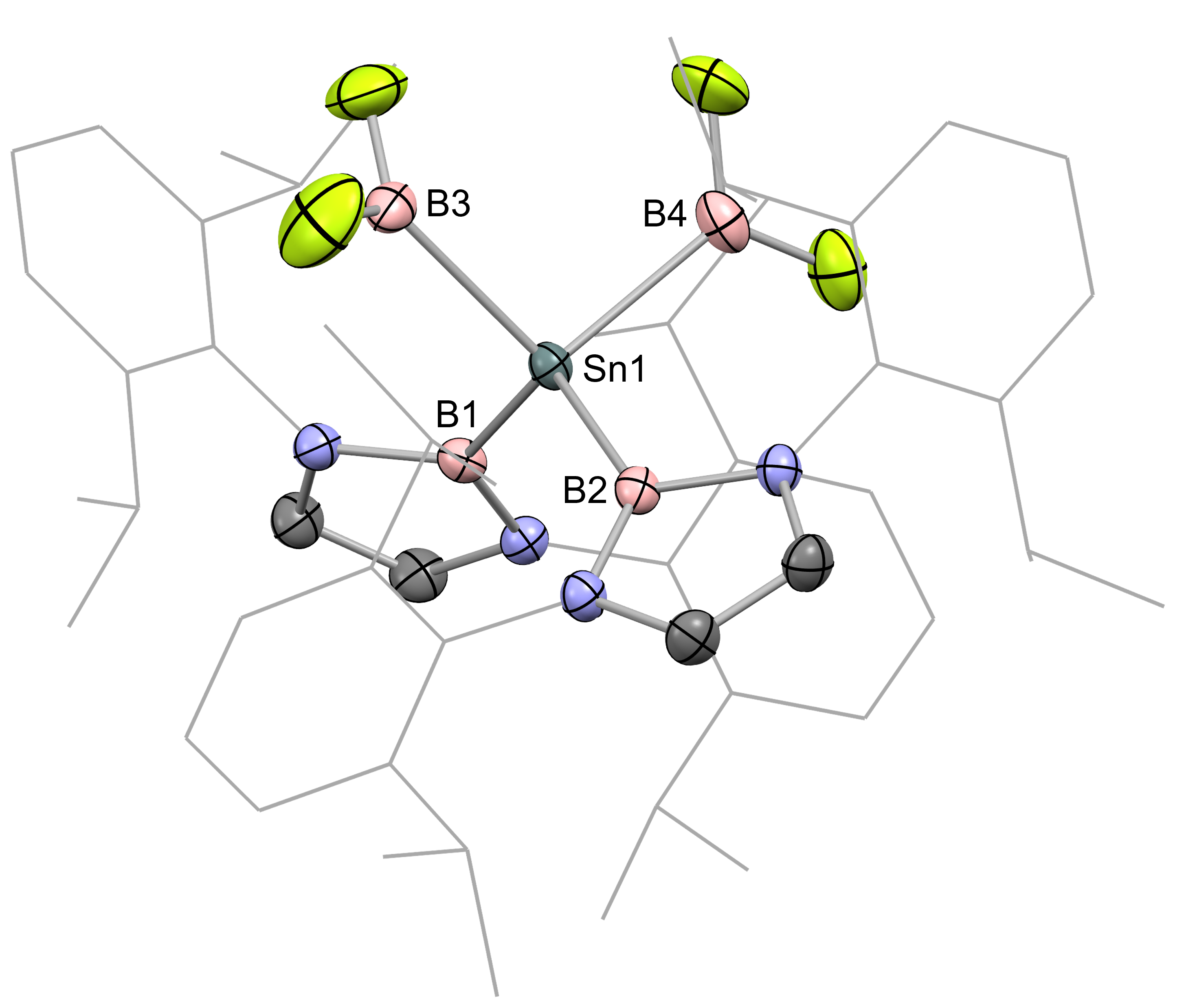


**Figure S18.** Solid-state structure of **2**. Atomic displacement ellipsoids represented at 50%. Ellipsoids of ligand periphery and hydrogen atoms omitted for clarity.

-----------------------------------------------------------------------------------------------------------------

**Crystal data for 3:** C_68_H_88_B_2_Be_2_N_4_Sn, *M*_r_ = 1119.75, colourless block, 0.200×0.120×0.050 mm^3^, hexagonal space group *P*6_5_, *a* = 13.25330(10) Å, *b* = 13.25330(10) Å, *c* = 61.0426(3) Å, *V* = 9285.63(15) Å^3^, *Z* = 6, *ρ_calcd_* = 1.201 g·cm^–3^, *μ* = 3.591 mm^–1^, *F*(000) = 3552, *T* = 100(2) K, *R_1_* = 0.0180, *wR_2_* = 0.0454, Flack parameter = –0.0234(11), 12062 independent reflections [2*θ*≤144.246°] and 710 parameters.


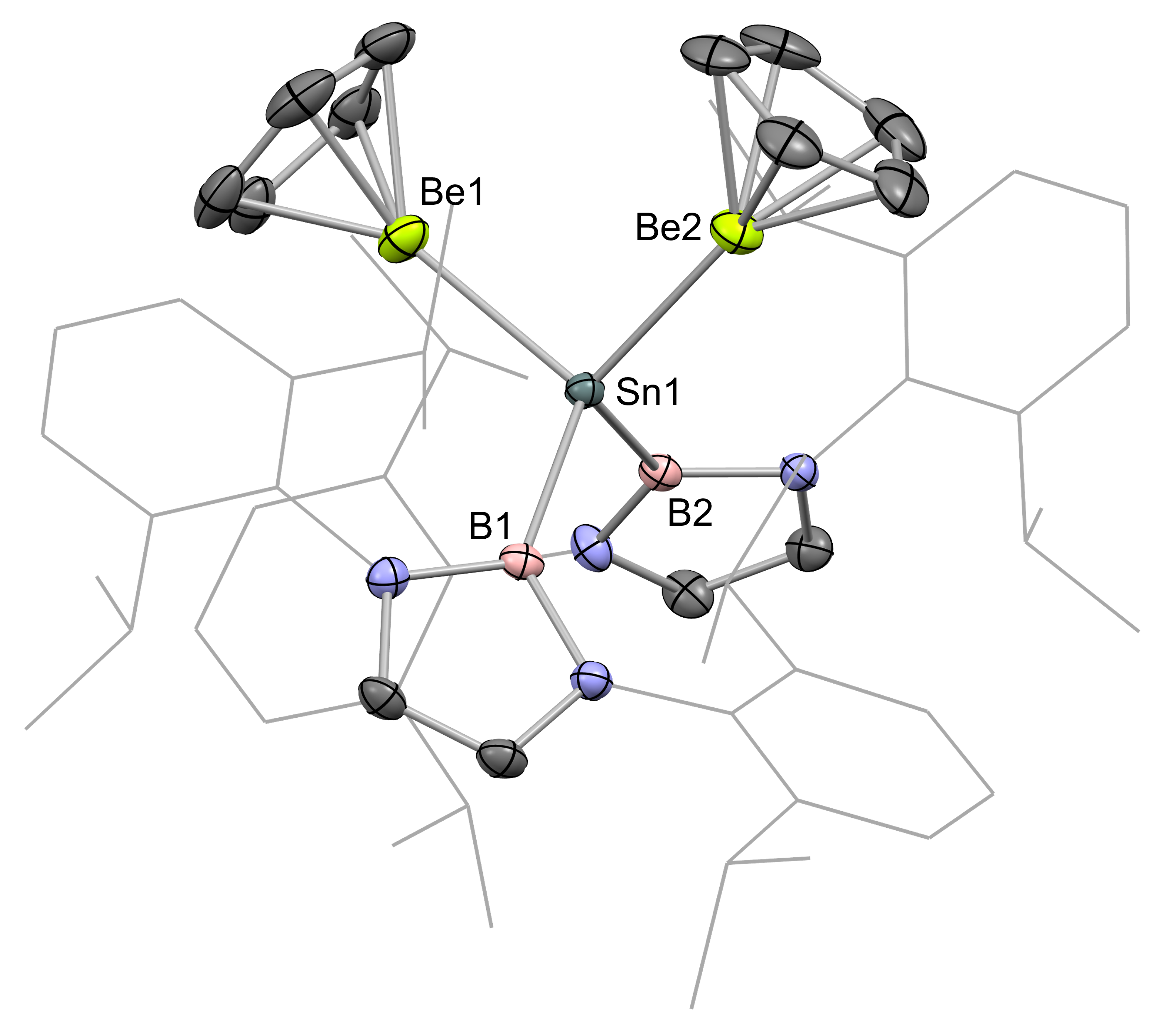


**Figure S19.** Solid-state structure of **3**. Atomic displacement ellipsoids represented at 50%. Ellipsoids of ligand periphery and hydrogen atoms omitted for clarity.-----------------------------------------------------------------------------------------------------------------

**Refinement details for 4:** The structure showed a mixed hexane/benzene disorder.The atomic displacement parameters (ADPs) of overlapping atoms from different PARTs (RESIs 8/9/10) were restrained using similarity restraint (SIMU) and rigid body restraint (RIGU). The 1-2 distances within the disordered hexane moieties were restrained to the same length using SADI.

**Crystal data for 4:** C_58_H_89_._25_B_5_N_7_Sn, *M*_r_ = 1057.34, colourless plate, 0.267×0.165×0.025 mm^3^, triclinic space group *P*‾1, *a* = 12.04710(10) Å, *b* = 12.99270(10) Å, *c* = 20.8514(2) Å, *α* = 76.5290(10)°, *β* = 74.3380(10)°, *γ* = 74.3860(10)°, *V* = 2981.05(5) Å^3^, *Z* = 2, *ρ_calcd_* = 1.178 g·cm^–3^, *μ* = 3.710 mm^–1^, *F*(000) = 1122, *T* = 100(2) K, *R_1_* = 0.0320, *wR_2_* = 0.0806, 11328 independent reflections [2*θ*≤140.142°] and 759 parameters.


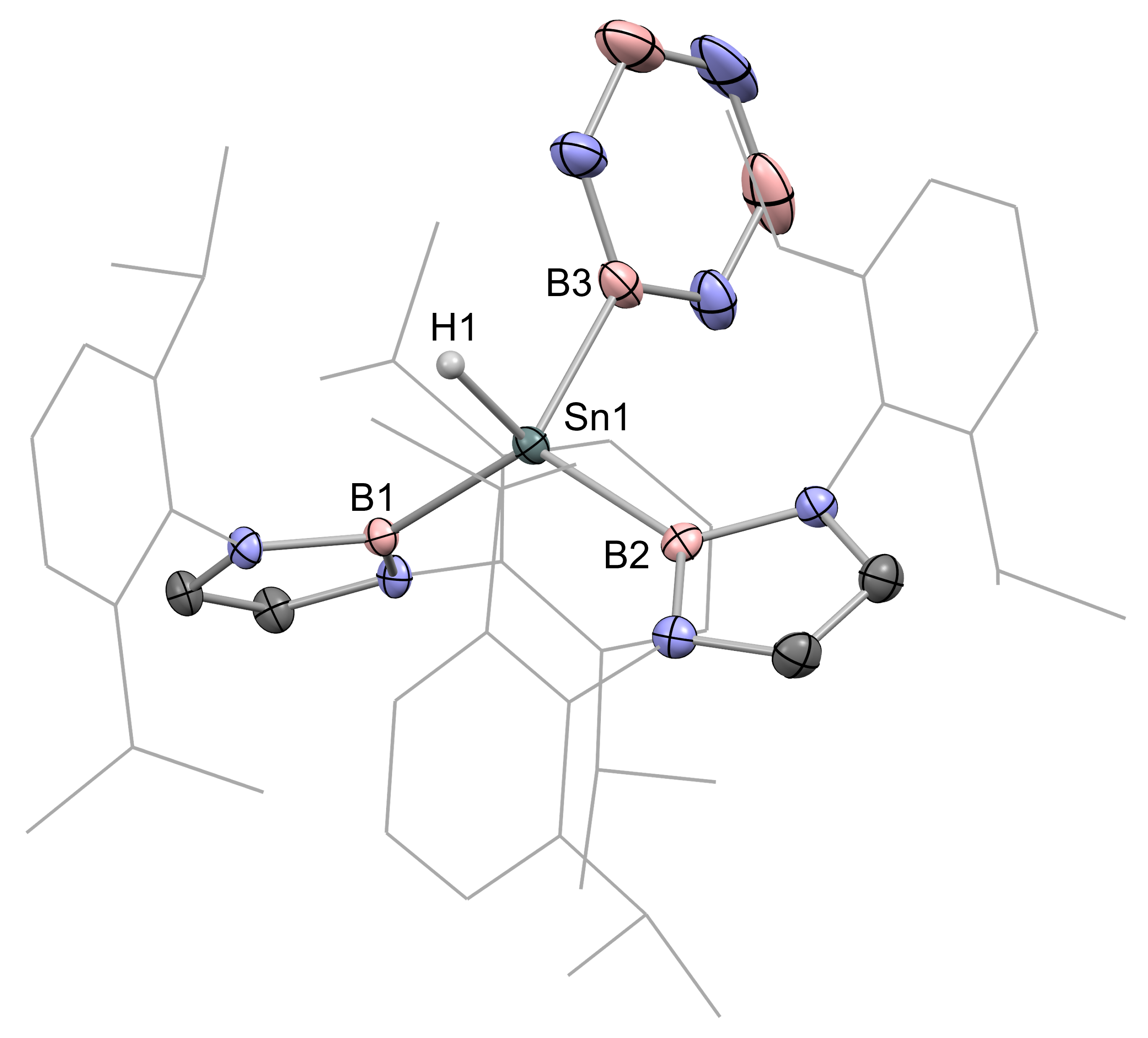


**Figure S20.** Solid-state structure of **4**. Atomic displacement ellipsoids represented at 50%. Ellipsoids of ligand periphery and hydrogen atoms except Sn–H omitted for clarity.

# Computational details

The structures of complexes **1** – **6** were optimised using ORCA (Revision 6.0.0).^[11,12]^ Specifically, structures were optimised with the ωB97X range-separated hybrid, in conjunction with the Def2-TZVP basis set, reparametrized D4 dispersion correction, and CPCM solvent (benzene) modelling.^[13–16]^ The nature of the stationary points (minima) was confirmed by full frequency calculations, and are characterized by zero imaginary frequencies. Natural Bond Orbital and Natural Population Analysis calculations were performed on the ORCA wavefunction using NBO 7.0.^[17]^ Quantum Theory of Atoms in Molecules calculations were performed using the ORCA wavefunctions for the respective complexes, and were generated using Multiwfn 3.8.^[18]^

Complex **2**: HOMO–LUMO gap = 9.61 eV


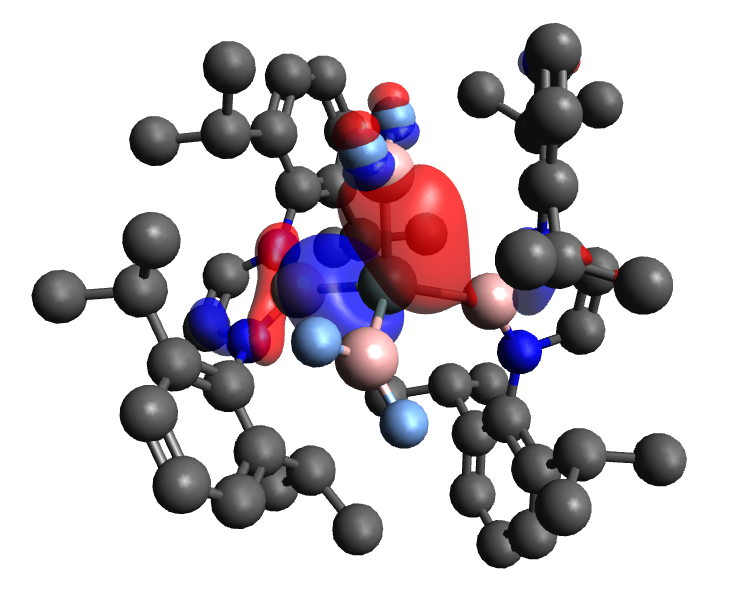


**Figure S21.** HOMO–10 of complex **2** (isovalue: 0.05 a.u.).


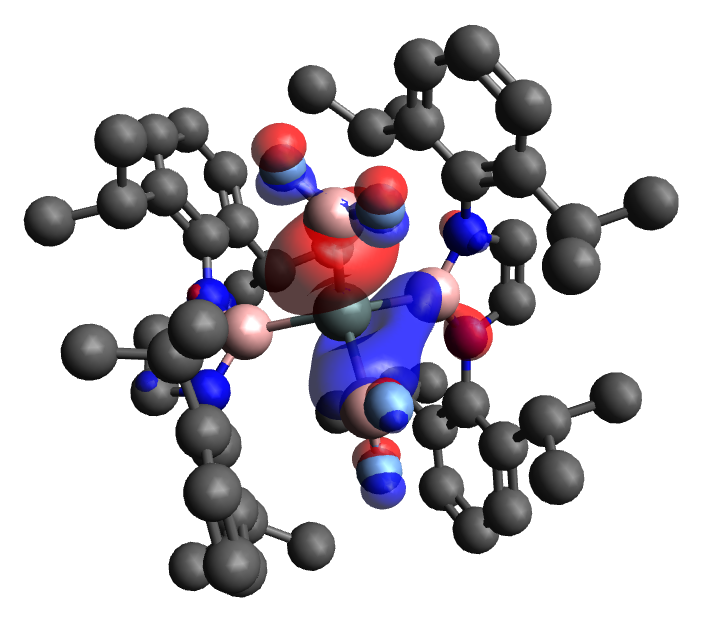


**Figure S22.** HOMO–11 of complex **2** (isovalue: 0.05 a.u.).


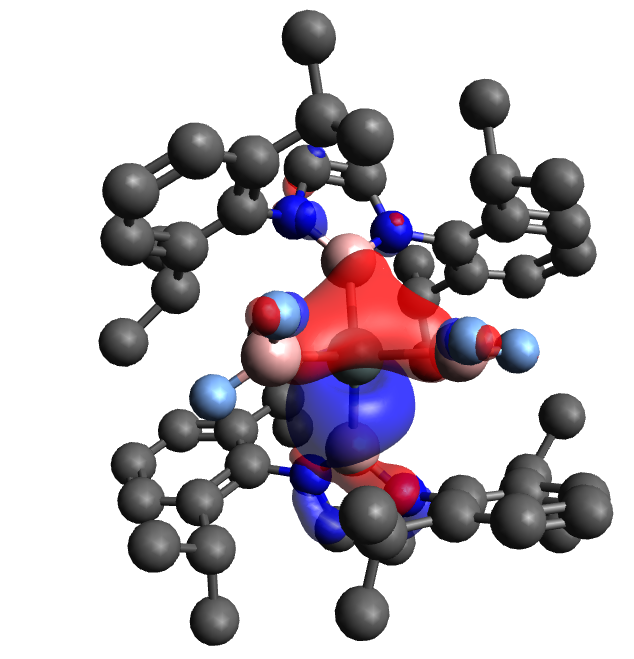


**Figure S23.** HOMO–12 of complex **2** (isovalue: 0.05 a.u.).

Complex **3**: HOMO-LUMO gap = 8.82 eV


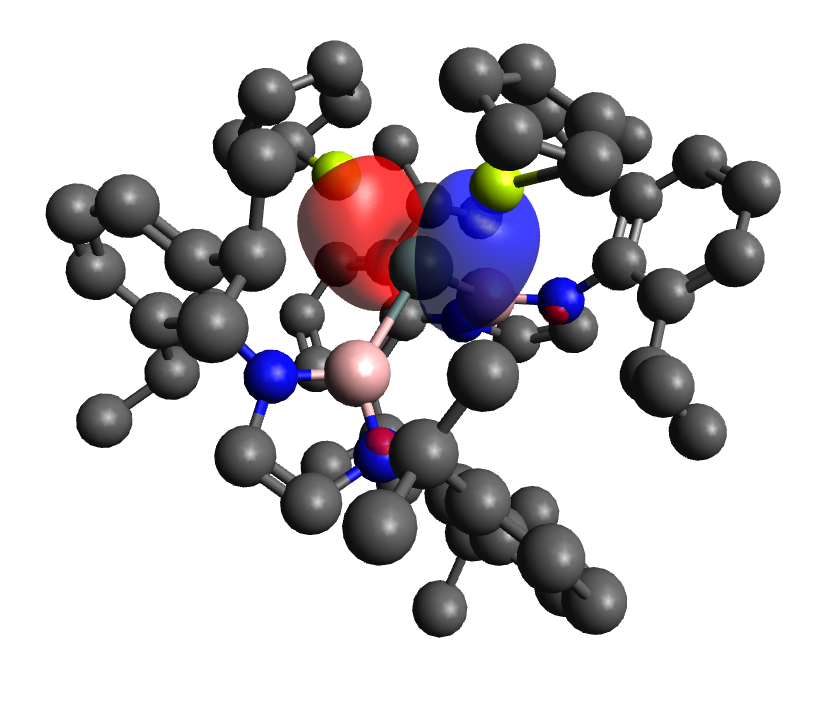


**Figure S24.** HOMO of complex **3** (isovalue: 0.05 a.u.).


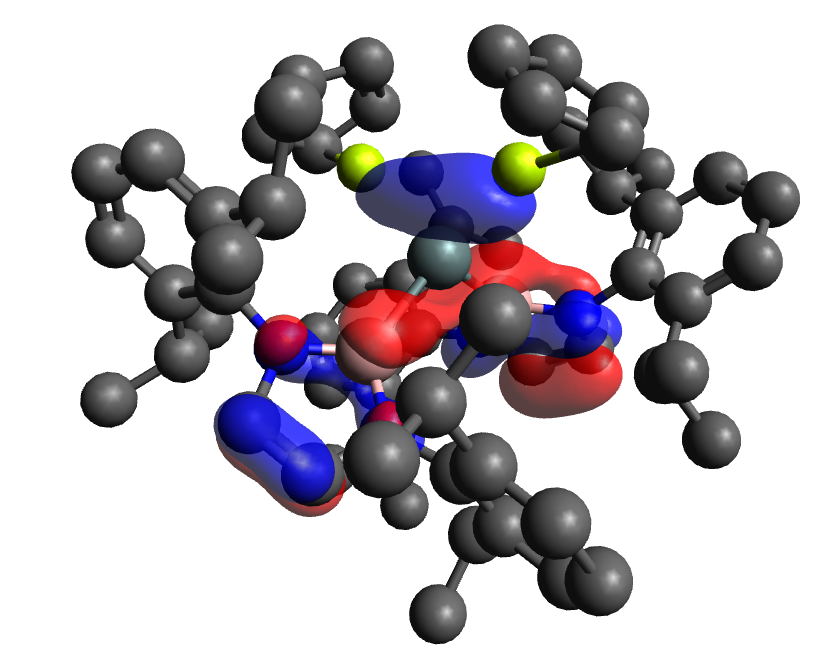


**Figure S25.** HOMO–1 of complex **3** (isovalue: 0.05 a.u.).


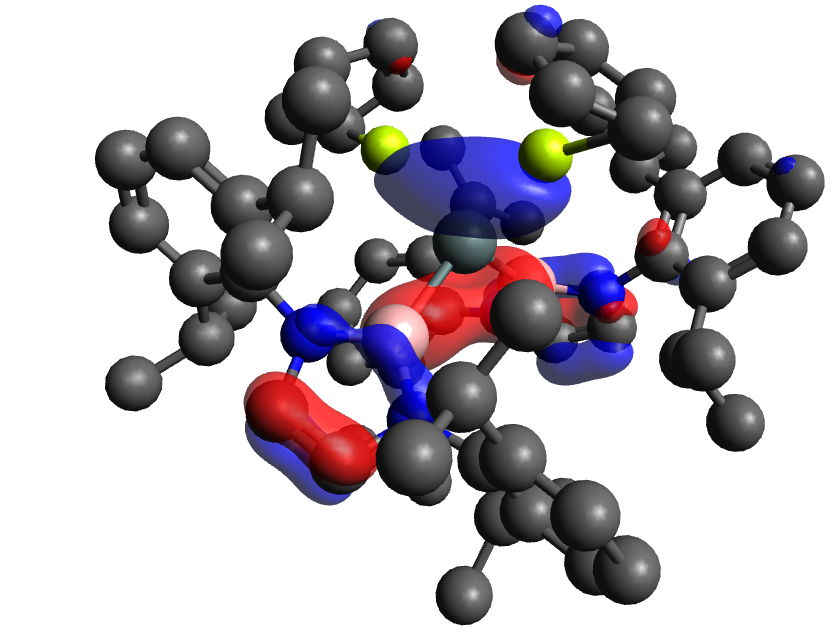


**Figure S26.** HOMO–3 of complex **3** (isovalue: 0.05 a.u.).


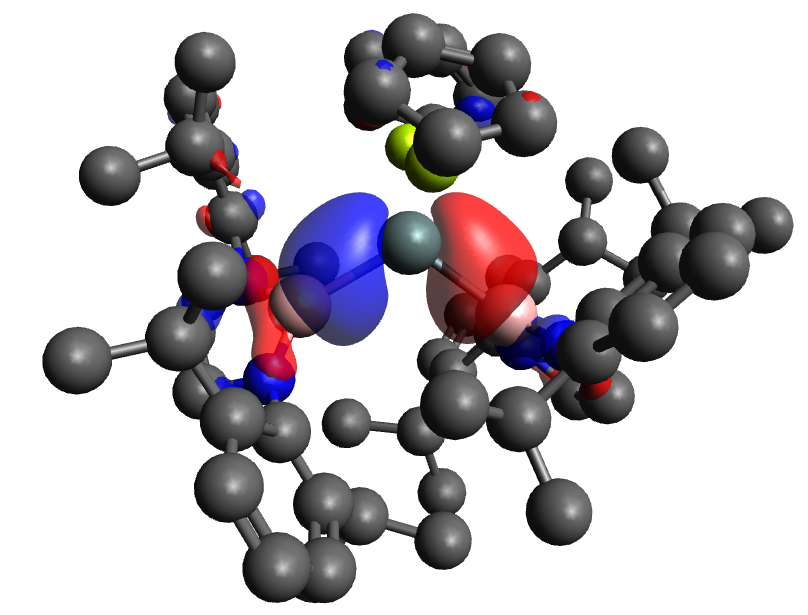


**Figure S27.** HOMO–4 of complex **3** (isovalue: 0.05 a.u.).

Complex **4**: HOMO–LUMO gap = 9.53 eV


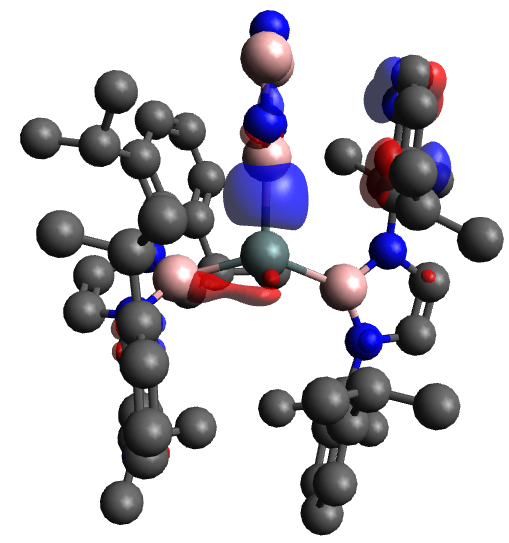


**Figure S28.** HOMO–2 of complex **4** (isovalue: 0.05 a.u.).


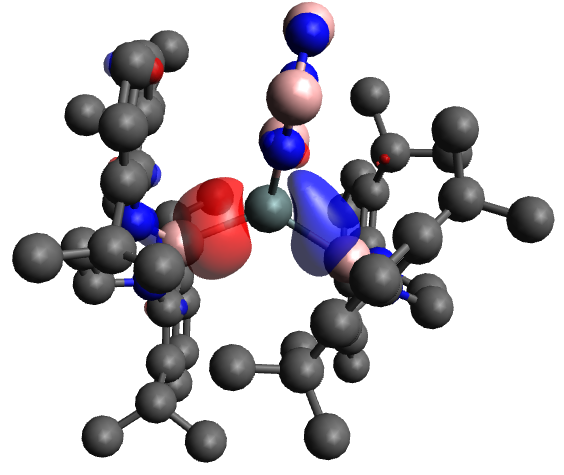


**Figure S29.** HOMO–11 of complex **4** (isovalue: 0.05 a.u.).


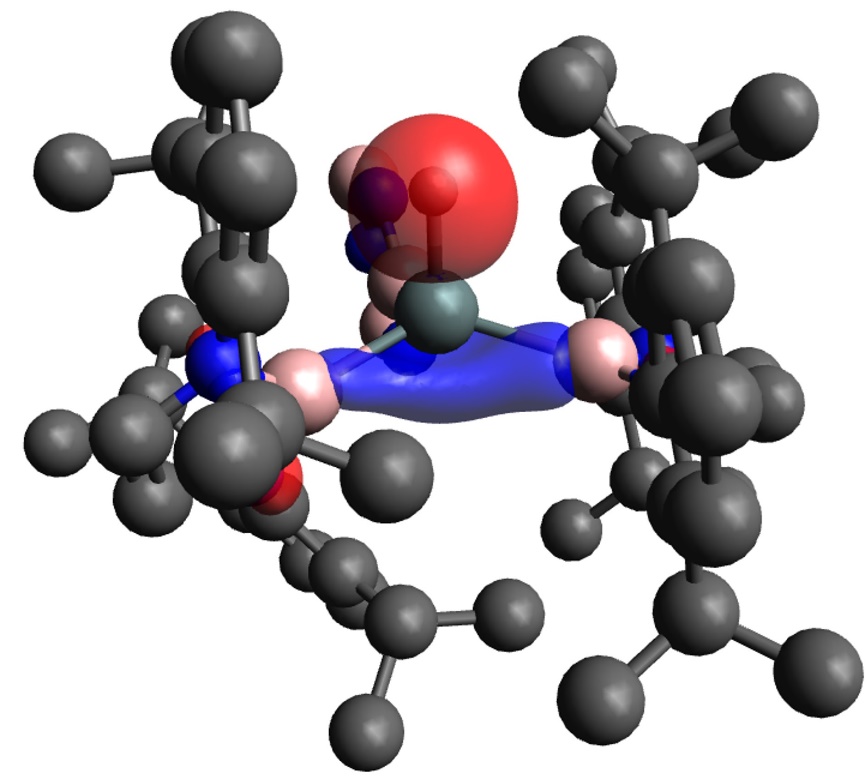


**Figure S30.** HOMO–12 of complex **4** (isovalue: 0.05 a.u.).

# Coordinates of Optimized Structures

Complex **1**

Sn 2.69099256163649 11.22504508222938 3.44936298003161

B 2.65735450188090 9.24198334461517 4.58595122686092

N 1.59436470977501 8.55844271258016 5.28152277479521

C 1.88894450881833 7.19274092906210 5.34093322712018

C 3.06743349543028 6.96604323641829 4.73786070504423

N 3.56774172461901 8.17556991343890 4.25756166711722

C 4.80997778210484 8.22199232145392 3.56250274370363

C 5.99718398611162 8.22423540804808 4.30157713465902

C 7.20600567029994 8.24045934415894 3.61155526227611

C 7.23275425524409 8.25436596028578 2.22733081967382

C 6.04559701131828 8.25283839884531 1.51112083268289

C 4.81552913690859 8.23025863614544 2.16073370797930

C 0.35768809100092 9.01767289729938 5.82132644054419

C -0.67787937740797 9.39413730219603 4.95755931511306

C -1.90259200199871 9.75904682452501 5.51142616085609

C -2.10539148082517 9.72945339647351 6.88058858384700

C -1.06903858016870 9.35591463114245 7.72089546180521

C 0.17648733196651 9.00473660185299 7.21200765356159

B 3.12126640293719 13.05374136660735 4.74368847041605

N 4.25596962253127 13.65434403162676 5.39450800426794

C 5.55131021499068 13.16968361765346 5.74063658490169

C 6.50429817276501 12.96150102809325 4.73364552571315

C 7.80085810278174 12.62833933326248 5.10809487537563

C 8.14925693240652 12.51220279758190 6.44507056698405

C 7.18811183771812 12.68630879629344 7.42482548729757

C 5.87441053542799 13.00510943483695 7.09180457504226

C 4.83111965266743 13.13047657939275 8.18700912137113

C 5.16032908915538 14.26691173623742 9.15826563607978

C 4.67980622608763 11.79949762513502 8.93035211984050

C 3.97161284530492 15.00029027351090 5.65637923692213

C 2.73116229142376 15.28637578144016 5.22646774820834

N 2.17601631775675 14.13856525299987 4.66651322516281

C 0.87085486977298 14.13221024329465 4.09312696885751

C 0.73420999769315 14.24535362491777 2.70397960733831

C -0.55168918405909 14.23077178437174 2.16870717434157

C -1.66145230550155 14.12242463679491 2.98803677064350

C -1.50746920670196 14.03174505789646 4.36343171273282

C -0.24269796265436 14.03746086354761 4.94143153986184

C -0.05626533361967 13.95420834134498 6.44761724571693

C -1.28983144521082 14.40150371458786 7.23446072143397

C 0.34582536983127 12.53954594701170 6.87521327250175

C 1.93770155559035 14.47492101987235 1.80937578589577

C 2.16163236765880 15.98095064974477 1.62510621528595

C 1.82833030689861 13.76833900691525 0.45825244316840

C 5.99254659876868 8.19067853071926 5.81825023347725

C 6.69260043201596 9.42019079742642 6.39659269374621

C 6.62078862236257 6.89374775959591 6.33747649699370

C 3.52738591371529 8.12792286529031 1.36439159081446

C 3.25912523132398 6.66217104624747 1.00202371332276

C 3.52227885031410 9.00943438248493 0.11482145504965

C 1.28932760456782 8.60952387856340 8.16698898313467

C 0.96224730388067 7.29581321380796 8.88469633485245

C 1.57659343389026 9.72021772742576 9.18104175986072

C 6.11281357643648 13.02701214999900 3.27147491105434

C 5.80290356709827 11.60969954813574 2.78085526398825

C 7.16654791120963 13.68397339353576 2.37960292222359

C -0.52728120470463 9.37018580088559 3.44809906403064

C -1.39779358885498 8.26964325401510 2.83259802707738

C -0.84514089895464 10.73515734233405 2.83483080090975

H 1.21729829100194 6.49244983804680 5.81182621828943

H 3.60312486742263 6.03819322565007 4.60820941746940

H 8.13860083517386 8.24138768294767 4.16570751479141

H 8.18211678233621 8.26498535412281 1.70183069482259

H 6.07941576874204 8.25594213189181 0.42782938458210

H -2.71565329899829 10.05365000911710 4.85666766661121

H -3.07034958458209 10.00247280352241 7.29450834111484

H -1.23008312208969 9.34429352396982 8.79356396146198

H 8.55421461023648 12.46400735437643 4.34637162529086

H 9.16901306343586 12.26551595186845 6.72112630987753

H 7.46034501664482 12.56474332866998 8.46791512957632

H 3.87324028228149 13.35641985126346 7.71618074127601

H 4.37740621168472 14.36020539323527 9.91613687512054

H 5.24433072339814 15.22329249415892 8.63640601999819

H 6.10629796084363 14.08435317850579 9.67626961700011

H 3.89285206807244 11.86934913120179 9.68598034009000

H 5.60781748351105 11.52133104302411 9.43791455010214

H 4.41937798181974 10.99444052069232 8.23910750806099

H 4.69745398992534 15.64409576077194 6.12807264177731

H 2.19037464744496 16.21938754604869 5.27307128520777

H -0.68748302503617 14.31508906004724 1.09706757782415

H -2.65583885407895 14.11455729581794 2.55387127050778

H -2.38720497187104 13.96204177423588 4.99087469404015

H 0.76331557722243 14.62780194984560 6.71115394398547

H -1.04594200445517 14.46296001161028 8.29809159735392

H -1.64704379598118 15.38253930387979 6.91047454563662

H -2.11143601646548 13.68656257543408 7.13358817850436

H 0.48076885673005 12.49685868686336 7.95949742264466

H 1.27778361918112 12.22176446592964 6.40617835445732

H -0.43285351072548 11.82534765431677 6.60518335002487

H 2.81272305083295 14.07079158296270 2.31972856401646

H 3.04771585847381 16.16533684832683 1.01073113477804

H 2.30521378227219 16.47886668019417 2.58647923285567

H 1.30142128257513 16.44074943624909 1.12901568929539

H 2.77407653724121 13.85864580277825 -0.08336939350913

H 1.05126904706881 14.20945926657812 -0.17216985755703

H 1.61430821172310 12.70564300831739 0.59060079964438

H 4.95402351402362 8.21493482822271 6.15131775049447

H 6.64484030681518 9.41135697515812 7.48902894598559

H 6.22463698988555 10.34106800981780 6.04601052649537

H 7.74682567488017 9.45414298002642 6.11022079153688

H 6.58919308735100 6.86438266556523 7.43033268562324

H 7.66732119641363 6.80990018715392 6.03052752891652

H 6.08964187902854 6.01718805370501 5.95743342259152

H 2.71140119152309 8.46404226424069 2.00526865954597

H 2.31576365411426 6.56758943314121 0.45655002588250

H 4.06021553002078 6.26779942827741 0.36941028180438

H 3.19738746131649 6.04068979573075 1.89819278970876

H 2.53349958819842 8.98515327110585 -0.35095929773819

H 3.74819383328593 10.04864900837903 0.36420628390748

H 4.24274170579077 8.66489555440154 -0.63211026817418

H 2.19714278023359 8.45721213881760 7.58143831451056

H 1.78950199426924 6.99913602915430 9.53571057762123

H 0.06741839084052 7.40081773343862 9.50510433151511

H 0.78218781228152 6.48557271625091 8.17474517866114

H 2.41911206846458 9.44047746581934 9.81986064207100

H 0.71496747453486 9.90415323084161 9.82882777923134

H 1.82751190862768 10.65438360210868 8.67675013153394

H 5.20160246654873 13.62391287408898 3.19850823324776

H 5.32077881903736 11.61326307761894 1.80140379464150

H 6.71239760568192 11.00881668853114 2.71156902492561

H 5.17636314829875 11.07060621845346 3.50569496650859

H 6.78853383975142 13.77072958049935 1.35725235718734

H 7.41765361253296 14.68536372984667 2.73723123111191

H 8.08758862864630 13.09723070475730 2.33548123859692

H 0.50958771482690 9.12873091312720 3.21257851563347

H -1.25340160732531 8.23010997743330 1.74910325783376

H -2.45896944990112 8.45279718484704 3.02471457657494

H -1.14416213744296 7.29109567675359 3.24789457631434

H -0.61142382275681 10.74306865565070 1.76742242831886

H -0.27378929608955 11.53811702941574 3.30753234699572

H -1.90187774015704 10.98971561493526 2.95071983979139

Complex **2**

Sn 5.8019534619475 7.90640480057372 3.63746040635692

F 6.84056018526418 9.62957716630759 1.18478869933194

C 9.35947184801975 8.84945083620305 6.73720654920234

N 7.62069613231935 7.16866367823017 6.45430920209983

B 3.95296011556897 9.04081174128959 4.13156115462613

N 7.22385630758212 5.41642371404729 5.06958497714832

N 3.84783576283220 10.46931943846404 4.04255858888346

N 2.65633554101559 8.60111739606618 4.55847232430455

C 4.66282540700075 11.35535656564077 3.26510452086402

C 5.93680066578457 8.49178709956836 8.44842363669741

H 5.65500523052492 7.71355350263140 7.73905860645935

C 7.30064511325251 9.01392777258962 8.04212358185111

C 7.94140895707040 4.96435974250606 6.17634957862231

H 8.22579347419882 3.92967845854228 6.28584350439971

C 6.98149714106375 4.55954176435980 3.95522031726008

C 2.48747087589440 7.13308820571487 7.07738006537927

H 3.09946040770324 8.00405407091999 6.84073240014052

C 8.17113491558855 6.00085485637219 6.99648450248874

H 8.69499372860101 6.02176620818452 7.93911375537592

C 1.14284109342205 5.44708856049993 5.79833346757944

H 1.00055181126253 4.91761494712047 6.73394849384426

C 1.88225406580662 6.62715696516576 5.78444980005608

C 6.08018373930222 3.50149625086339 4.08326471640094

C 9.14178627333128 10.52211670268846 8.46906308002567

H 9.56663045139940 11.34549100497993 9.03343222825528

C 7.85783870354708 10.08370834955650 8.73957231994198

H 7.28458510164577 10.56421638287640 9.52497173208362

C 1.50456807941906 6.79047129763407 3.37791000259125

C 8.06587244925496 8.39680072422887 7.04838650631529

C 2.03440037619598 7.30810344193597 4.57386826508752

C 4.86411860348470 9.58204959937333 8.40348044247658

H 3.90270683118547 9.18003169100441 8.73159326607060

H 4.73831100227573 9.97485766674697 7.39240011105304

H 5.11024391348695 10.41911531403562 9.06210860967020

C 5.80166847536186 11.94652985470404 3.82684147253165

C 0.59771497685379 4.93485402055149 4.63748256996706

H 0.02840462486558 4.01175216345531 4.66272347255832

C 2.56271171907066 10.84016859259317 4.44335672190425

H 2.25039562169500 11.87300969165492 4.46274249631596

C 7.67902153542798 4.79334086353607 2.75661211802393

C 1.63432549476042 7.52058148074690 2.05177654293821

H 2.56541886206680 8.09152078698221 2.07707158357341

C 0.79561971225352 5.59627503794668 3.43593866929490

H 0.38089353469847 5.17742914800399 2.52745233285700

C 7.41948669235600 3.95753840086270 1.67654376621291

H 7.92697638430227 4.12483320617461 0.73477261187869

C 4.29561375990217 11.59672597159769 1.93587110916934

C 9.87821643299839 9.91437790064479 7.46515145028789

H 10.88008205215135 10.26866531663924 7.25086950509461

C 1.86271626370706 9.73667154332588 4.76047216636596

H 0.84714184613992 9.65317148671148 5.11576591194596

C 6.01928539115108 7.85975849857926 9.84303748927933

H 5.04805535762492 7.46509589256259 10.15251629792914

H 6.32923101587823 8.59847629089995 10.58746855980242

H 6.74260751359034 7.04048205040423 9.86189336390602

C 5.87430667801629 2.67278821439095 2.98333780540182

H 5.17341643358343 1.84901029964421 3.06120887109636

C 6.56764294478821 12.78741721504078 3.02557030865199

H 7.46134797166435 13.24509827807793 3.42868042275384

C 8.75569474381743 5.86204510099521 2.65788176449230

H 8.47056032801972 6.69592712439096 3.29947725035389

C 6.52621191604507 2.90230109165679 1.78674657435166

H 6.34026798119378 2.25974310454356 0.93302754309010

C 5.27221748636790 12.55825197492237 6.19107604645159

H 5.54846058141242 12.41151928257803 7.23855356893695

H 4.21814363872825 12.29943988990093 6.07790521260345

H 5.39246028827751 13.61931985539662 5.95230492020100

C 5.30868392987511 3.24560374189655 5.36385464655593

H 5.55071779578691 4.04090805627058 6.07017778714236

C 3.39700124018028 6.07036969235318 7.70140390565852

H 3.83461353634638 6.43793895355109 8.63093101343262

H 4.21031060938375 5.80205499224809 7.02245420310595

H 2.84333564169564 5.15836713676831 7.93809373018400

C 3.79951849181244 3.29358238523702 5.11473641051077

H 3.25609250268061 3.18127564116510 6.05651653721042

H 3.47872501335505 2.48847524547277 4.44788275050703

H 3.50307826056231 4.23968419879821 4.66132604233898

C 1.69343198720248 6.58380881439633 0.84095076584972

H 1.99263018193769 7.14965094575242 -0.04462411714362

H 0.71375830332023 6.14718775372774 0.62723726996651

H 2.40509162621061 5.77049135160700 0.98207729642520

C 1.39757109810649 7.57175582383844 8.05996235705409

H 1.83975575999005 7.95357084647949 8.98467632531441

H 0.74528681698685 6.73420567405749 8.32321654768417

H 0.77416378328440 8.35994463533116 7.63064633288800

C 3.09591752838408 10.93220393432065 1.28743044513605

H 2.62092605449082 10.28786280652788 2.02488647009266

C 10.22600211441737 8.16884639180127 5.69102065286026

H 9.58212992735454 7.52236092318994 5.09329122088009

C 7.63565966303326 11.99201660740861 5.58332057671599

H 7.85891439134207 11.68289345636073 6.60186328111294

H 7.86387837789833 13.05929906658672 5.50690841978627

H 8.30036437514705 11.44809268344997 4.91040495943639

C 5.08048825878698 12.45908481399479 1.17692409769429

H 4.81245096755011 12.65408579579904 0.14435239549442

C 6.16775461839833 11.71054774033168 5.27885706580131

H 5.97995107370568 10.65667710361736 5.50654998511518

C 6.20928668538079 13.04920611647069 1.71276942426669

H 6.82097093938821 13.70750526309925 1.10522514778221

C 10.07920680443372 5.30911701981162 3.20188195276433

H 10.85776284508734 6.07680618363803 3.16945671362781

H 10.41210953829620 4.45903760195451 2.59907512436591

H 9.97519312756390 4.97454041408388 4.23612170292929

C 8.94213572450672 6.42726176802339 1.24947301483021

H 9.60953828103059 7.29240929308416 1.28786750270997

H 7.99466449918248 6.74670803728785 0.80943472804572

H 9.39824632098546 5.69715749625400 0.57539604141482

C 0.47263100835982 8.50739805751007 1.86759608366584

H 0.56325473285707 9.02864883487937 0.91079356790492

H 0.44128715669101 9.25586718955899 2.65927589691791

H -0.47853838605236 7.96662625760399 1.86796565277476

C 3.52864327505110 10.05512261811515 0.10857164108351

H 2.66144238777861 9.55774359192008 -0.33430344530792

H 4.24264071434659 9.28998671065520 0.42127821157603

H 4.00697918212745 10.64922777117314 -0.67451911915597

C 11.29444799973528 7.29036455613995 6.35353756075808

H 11.90211619333813 6.79288241800166 5.59234058997028

H 10.85018212996235 6.52094008246525 6.98577723250323

H 11.96028145687835 7.89889637782983 6.97297592668263

C 5.72154388402371 1.91337768503582 5.99786727663662

H 5.18743403493884 1.75547707083809 6.93910166020208

H 6.79426426650837 1.88404997386686 6.20565968976807

H 5.48929935028759 1.07451051404199 5.33565522086672

C 10.89391501947787 9.17107057038005 4.74435260217466

H 11.38701841671013 8.63625228238698 3.92753754701784

H 11.66190563338258 9.75601223825929 5.25778049843178

H 10.17106236318478 9.85983215593436 4.30955031750676

C 2.05565771737100 11.96927956983069 0.85445979954428

H 1.17727549672968 11.47569887905489 0.42959296363845

H 2.45860686001592 12.64588333705295 0.09574431346215

H 1.72799980135908 12.57481770466320 1.70333500221387

B 7.12188344017700 9.30231090568991 2.44100808502860

F 8.27539434846318 9.78782726528332 2.90207575720139

B 5.12337211652597 6.38676910672636 2.10156945944430

F 4.31280346422534 5.37769484507835 2.40562933911053

B 6.97073161042743 6.82347905689188 5.21630712527737

F 5.38411960576606 6.52322042004671 0.79974290238687

Complex **3**

Be 6.99722672905548 5.50280230897929 28.61634016354543

Be 5.33488548073372 7.10122722237554 31.32037155929219

Sn 5.30017756029696 5.00164866248922 30.19888847682204

B 3.36088720984737 4.44610337244478 29.14460326541478

N 2.07425835314024 3.93056991419383 29.59925701565765

C 1.27418833480875 3.66131321451328 28.48152331944976

H 0.27535144839526 3.26805099843411 28.59176517854888

C 1.93167310507498 3.99055599227348 27.36289851218757

H 1.60963417573964 3.91521031319512 26.33570834404487

N 3.19370849017531 4.49051723592576 27.69790847886673

C 1.33489810980702 4.00048229451033 30.82988430393252

C 1.21407543393012 2.88064981774241 31.65950538439384

C 0.30702475044837 2.92317386962157 32.71601642857723

H 0.19088574100374 2.05097446534768 33.34996126660120

C -0.47817736063940 4.03867629619793 32.94354278422510

H -1.19160990995893 4.04838831867137 33.76084855284807

C -0.34380584585344 5.14466575155697 32.12076631167342

H -0.95671265539726 6.02136369813934 32.30054357983310

C 0.55517264375902 5.14544474535767 31.06046779914966

C 1.93607199068602 1.58537970774490 31.35903415837345

H 2.65631499624251 1.80125787829729 30.57389712488624

C 0.92928933544049 0.55991181558901 30.82322479701622

H 0.16433460044394 0.33933526187114 31.57341824027265

H 1.42457134808629 -0.37970376939779 30.56584151533642

H 0.42442953002065 0.93533843696252 29.92945927602782

C 2.69398496016421 1.03176421952204 32.56714894120556

H 3.49707235850249 1.70260126167210 32.87542862010113

H 3.14458787133699 0.06688522485287 32.32378787812280

H 2.03360186714923 0.87512717473039 33.42373758579946

C 0.62413558141375 6.36205957917191 30.15836140929044

H 1.30798055463349 6.13630156241830 29.34282855312658

C 1.19095536200307 7.56553176007717 30.91415049183376

H 0.55870145624176 7.83896534574857 31.76407478284810

H 1.25902281198984 8.43608226112097 30.25418141018491

H 2.18971041689417 7.33867836656675 31.29092320867811

C -0.74249904458883 6.67708101306397 29.54410220572215

H -1.12131541649039 5.82215187300420 28.97876588513980

H -0.67043534208586 7.52886235403710 28.86301103272670

H -1.48027654281492 6.92674257289262 30.31134678099288

C 3.94947185832160 5.07185995713872 26.63045170531732

C 4.01745456614992 6.47041837752345 26.51143283154682

C 4.63150629482466 7.01290048508180 25.38779894514854

H 4.68484372530597 8.08882020261848 25.27400678341132

C 5.14929105137443 6.20549401353950 24.38727065077596

H 5.61290629811886 6.64977770441016 23.51280640593050

C 5.06594350599043 4.83176963837377 24.51205077123975

H 5.47385215475230 4.19742430286788 23.73216613107297

C 4.47658215177885 4.24623155227825 25.63036448799236

C 3.32358515316087 7.38887115833483 27.50097073268795

H 3.35263526601624 6.91783731439665 28.48528251801111

C 1.85873795903255 7.55797195381105 27.07751823917201

H 1.33133802264897 8.22281586827818 27.76639868486946

H 1.33375099108458 6.60204540352961 27.05547727505055

H 1.80860107821802 7.99754517208270 26.07677688875430

C 3.98516292821488 8.76061233724710 27.63178398113233

H 5.05054743592786 8.67122059421178 27.84917348857982

H 3.51712922752566 9.31931268645094 28.44551110830175

H 3.86794676351785 9.36125506743574 26.72599916901567

C 4.43384836803636 2.73555451717047 25.72231556475482

H 3.85410004132494 2.47047749833472 26.60187027614012

C 5.84520258438571 2.17280070716369 25.90667220311884

H 6.49832330865996 2.46416732301000 25.07804976077047

H 5.82174037162927 1.08226229460326 25.94514286922612

H 6.29083400102651 2.52863862234303 26.83872239025760

C 3.73884541747892 2.10861987517550 24.51070149946807

H 2.73720073165220 2.52329736170788 24.36951790017617

H 3.64230910194760 1.02812821846946 24.64945241177794

H 4.30224461437436 2.27500260277922 23.58834354521269

B 6.04653159032843 3.33325834936977 31.55005054400904

N 6.52027783991647 1.99102922131419 31.24193871542710

C 6.63365860428978 1.26667413258808 32.43360448015981

H 6.91171095463379 0.22387102512205 32.43144309747176

C 6.35618537691005 2.06451049572158 33.47456163866861

H 6.37345567495650 1.83912190569681 34.53008026521302

N 6.02365331185687 3.33867018273616 33.00383707488351

C 7.01930810164619 1.36432097836292 30.05059930075547

C 6.19706637510163 0.49012634288785 29.33438864405957

C 6.72556310228940 -0.19887726986379 28.24476391764751

H 6.09088710049789 -0.88208324878933 27.69073160834065

C 8.03884261815870 -0.02619917402082 27.86114452156432

H 8.43713560965575 -0.56318694349568 27.00664960921112

C 8.85340320837943 0.82915158264377 28.58731344834817

H 9.89009935408757 0.93882898864604 28.29409338177058

C 8.37565173751690 1.52105633162335 29.69517722737430

C 4.76073251184990 0.22401771909203 29.73029290609012

H 4.54222540900726 0.84030924022659 30.60006422519404

C 4.57353003759981 -1.24270341338395 30.13152187282774

H 4.77450255853370 -1.91411513813989 29.29208193675078

H 3.54773375741735 -1.42397457282702 30.46237061297742

H 5.24768130542044 -1.51721419078131 30.94726473468000

C 3.80416271780354 0.62674564389678 28.60619859183909

H 3.82726661527526 1.70769727674961 28.46239830257993

H 2.77747772723169 0.34094977183780 28.83854883236162

H 4.07435033274849 0.14920941789647 27.66067141387781

C 9.34548881172973 2.33617679060821 30.53597709264725

H 8.80544856363499 3.21124156551713 30.90311720495385

C 10.58734702832307 2.80856111645895 29.77181721311990

H 11.27738231032978 1.97878563712458 29.59559687438415

H 11.12894099904462 3.54431915411105 30.37297870182701

H 10.35896897034671 3.26096611174599 28.80815656135016

C 9.83915583922698 1.51552629665202 31.73815011464263

H 9.03619865854759 1.24438664143954 32.41902435459783

H 10.58311139549797 2.08388357556411 32.30304953527069

H 10.31823698003314 0.59710182215772 31.38543421727446

C 6.10741052882717 4.40987727154887 33.95092580649929

C 7.38291300212884 4.86710269845843 34.32223240328635

C 7.48733049828706 5.80536939442386 35.34358514379386

H 8.46437662094850 6.16647300242658 35.64327932934937

C 6.35775831874794 6.28161564611187 35.99130965635590

H 6.45364363051572 7.00419640346864 36.79499326862111

C 5.10763482294723 5.83667518835911 35.59998475399710

H 4.22809381193527 6.21128807840989 36.11041413372135

C 4.96045321654588 4.90124483567586 34.57712734111676

C 8.63494323983687 4.38365073972949 33.61410006457609

H 8.39495163020544 3.44609187616771 33.11526641234335

C 9.79307207440558 4.10485106528148 34.57346750483233

H 10.16536914809690 5.01819332891919 35.04473431338825

H 10.62963144464374 3.65776383564624 34.03089374887442

H 9.49305087228025 3.41320754183780 35.36443015526157

C 9.04500669648741 5.39702447492241 32.54090039858612

H 8.24426194711899 5.53374435016156 31.80963990124283

H 9.94423043002357 5.06078488665925 32.01582090003078

H 9.26602801227954 6.36847642687757 32.99467010830891

C 3.58388511540477 4.38820335411950 34.21869525148385

H 3.65597138101193 3.89469892476508 33.24894940970073

C 2.56595975766681 5.51664086155730 34.08245914811486

H 2.43170564393634 6.06511928511613 35.01901020122214

H 1.59882581147658 5.11084578744903 33.79931251020044

H 2.87412286217641 6.21969159089016 33.30819949433411

C 3.12427555780768 3.35859965975338 35.25592344102258

H 3.81732549118131 2.51527961576580 35.31215293950106

H 2.13523102268251 2.97297794842553 34.99863124325366

H 3.06590184828047 3.81435108559141 36.24932349042147

C 8.91066440007345 5.58360126340257 28.70329375198465

H 9.50765534312101 5.27365269346744 29.54471641910089

C 8.35636195062020 6.87064793268678 28.51789479054166

H 8.46662129118737 7.70781825809473 29.19006939333883

C 7.55204411711285 6.83853597023630 27.35472292437964

H 6.94632190652024 7.64341997835086 26.97077398084300

C 7.61330698357486 5.53057968284833 26.81670962727902

H 7.05273341406624 5.17524007906947 25.96903138658895

C 8.45113442647183 4.75300998282454 27.65006659354576

H 8.62233628778823 3.69202783173154 27.54997054679102

C 4.69054911469798 8.36391465010751 32.60132409450753

H 3.84904247538952 8.21843251629890 33.26038339885055

C 4.64062974092090 8.89104307660193 31.28609796973034

H 3.75156111270121 9.21313607826251 30.76792430363070

C 5.94755985995170 8.84142139695887 30.74718060168203

H 6.22487258531658 9.10743684907865 29.73944971366555

C 6.80142314533868 8.28004696630328 31.72422102920168

H 7.84528675076267 8.04016519134099 31.59380722399042

C 6.02634869674726 7.98259897377043 32.86995720289585

H 6.37433340780359 7.48261290126943 33.75842595817708

Complex **4**

Sn 10.19697419874085 6.00772141120513 15.86756623434877

H 10.01947686221152 4.79880269381150 17.08058075084038

B 11.00066222285111 7.76543782500769 17.02517711782012

N 11.43386573461131 8.99334137794997 16.43808141419353

H 11.37211332526842 9.10277626548394 15.43931706694989

B 11.96124915561249 10.08261776310009 17.19228754727176

H 12.33135615161753 11.09092646350620 16.65912462070194

N 12.01375604585265 9.93473330826331 18.61590453032480

H 12.38133568143769 10.69080540733873 19.16859662992803

B 11.56673670490329 8.75152349176680 19.27721119121995

H 11.62548459135622 8.64176569894935 20.47096968855335

N 11.04503179940173 7.70181254018164 18.46100435426888

H 10.71801079223645 6.86163590945145 18.90882545069542

B 11.77777202953025 5.18465138279948 14.50116374406839

N 13.13088581642807 5.68125987875257 14.51637034097449

C 13.90440250766497 4.91001454737725 13.64755565648263

H 14.95602032131939 5.10016066948663 13.50358892464257

C 13.13556738729635 3.95340577200607 13.10518249651024

H 13.40740202748945 3.17440066271647 12.41054605280459

N 11.82860974298787 4.06888528711560 13.59101453199154

C 13.75143484442638 6.66361031600935 15.35023413987972

C 14.21554861129656 7.85523528553034 14.78634054326497

C 14.82414889603708 8.79121242839216 15.61965116449287

H 15.18644052111756 9.72370975173597 15.20168430291995

C 14.96153142808054 8.55570538849909 16.97329288608907

H 15.41853803358235 9.30267147825584 17.61344045910663

C 14.52036159181475 7.35732949575150 17.51328361441140

H 14.64833530947654 7.17998802184847 18.57347931106012

C 13.92943069868824 6.38386820922668 16.71816684164128

C 14.10956129492216 8.15188012939092 13.30113204472733

H 13.48351041809299 7.38442594179282 12.84409376628289

C 15.49272394253591 8.09098321773904 12.64192896639604

H 16.15518310516252 8.85383676537320 13.06118571312615

H 15.41447972635610 8.26708041180671 11.56525994375429

H 15.96857568401135 7.11968493836026 12.79434498453825

C 13.45717618075851 9.51100715352651 13.03453315183719

H 12.47181035374807 9.58181196147527 13.49654731195265

H 13.33563126908632 9.66633209387049 11.95871211896058

H 14.06720373422133 10.33251841146598 13.41848123185440

C 13.56583724430507 5.02792934583226 17.30498179547317

H 12.60062932972379 4.72661243600819 16.89581928440169

C 13.42792322842173 5.02368279650256 18.82755328722008

H 14.39908683935073 5.13241746924486 19.31941866427757

H 13.00679653630639 4.06767548577614 19.14940039590339

H 12.77536823060324 5.82188220709715 19.18194930033843

C 14.60229020153515 3.97544637753510 16.88692127840770

H 14.63082790371501 3.83720429661850 15.80659179256034

H 14.36826688897527 3.01025063849972 17.34367645405330

H 15.59914667658998 4.27596126357016 17.22364608188323

C 10.91606469797099 3.01433976238273 13.26833362713071

C 10.61349220093713 2.04554753870124 14.23642166229503

C 9.85032355413023 0.94540471794005 13.85525193905572

H 9.61682799677329 0.18290155207725 14.58942954953302

C 9.40898350223967 0.79287477052084 12.55169383667014

H 8.82756059867004 -0.07884975381367 12.27054950627944

C 9.70733612112658 1.76343505939876 11.61197572188860

H 9.35212068293583 1.64697341434978 10.59372804773066

C 10.45465240607730 2.88720932456132 11.95105529010936

C 11.16610964052167 2.10139640784986 15.64816515871030

H 11.53305889206010 3.10987020930453 15.82949735832862

C 12.35552158277077 1.14311365490304 15.77658179886980

H 12.04766071869116 0.11440401983305 15.56625770678679

H 12.76649876757675 1.17279435507425 16.78947461667518

H 13.15213302675742 1.40809553079520 15.07683196390210

C 10.09985330460269 1.80944888480429 16.70618389653481

H 9.22271905011168 2.44487033038496 16.57505053899533

H 10.50480863377150 1.99839247258078 17.70426807771996

H 9.77300681815777 0.76613948281602 16.67815404909394

C 10.76325676749583 3.91357939981912 10.87619436413076

H 11.34547623615816 4.71466407113003 11.33275127417645

C 9.47671538573038 4.52729043050724 10.31422278851864

H 8.83894612866114 3.76474104770510 9.85911202348278

H 9.71090889991848 5.26609307087690 9.54216277323514

H 8.90047419591595 5.02109454320115 11.09888384410532

C 11.60592901751471 3.30005934182834 9.75243089579607

H 12.52885611772451 2.86199790586079 10.13961157908293

H 11.87367314083490 4.06165255895526 9.01485481223472

H 11.05696124037303 2.51000698094878 9.23247893358223

B 8.10905841847909 6.54679953952646 15.29000474347362

N 6.90001322790931 5.83831194147387 15.59537593827405

C 5.80965544067071 6.60727926784259 15.18533822611915

H 4.79365303658700 6.26535573603628 15.30910898294578

C 6.25676078060969 7.75848480661014 14.65385600536608

H 5.69605324201212 8.57975182421184 14.23646565790751

N 7.65228527361476 7.77508388566448 14.70817000028448

C 6.73603091163502 4.59733023996208 16.27852393172531

C 6.30333671101773 3.47559825461043 15.55966515908735

C 6.16277911260339 2.27307373148399 16.24548024548772

H 5.83523166346103 1.38591774938639 15.71833905681935

C 6.43774796941794 2.19202894158521 17.60104305281405

H 6.32849494794601 1.24530900588241 18.11964768519384

C 6.84689515820618 3.31722682714590 18.29739113829450

H 7.04818800926527 3.23723403170122 19.35847580256629

C 6.99882800710354 4.54177123817876 17.65397338124396

C 6.01279520826187 3.57838708455563 14.07067933083778

H 5.47233209978573 4.51261348737557 13.90244310148127

C 5.13165875247197 2.44634718311102 13.54150727058349

H 5.66013803546458 1.48863370277089 13.54368221393203

H 4.84890892919479 2.65666249955947 12.50684385975199

H 4.21571354104023 2.33356259163471 14.12745435567877

C 7.31390540875078 3.64333648745708 13.26783848990870

H 7.91838322836672 4.50790912627648 13.54277969452816

H 7.09778740442079 3.70422369322298 12.19808637286141

H 7.90827048339407 2.74752787753394 13.44247339800665

C 7.36681362705032 5.79121703562855 18.43805844533831

H 7.97546753516513 6.42797534440943 17.79455112848680

C 8.19006338862399 5.50368141121415 19.69275572863649

H 7.60269262799911 4.99021038000788 20.45885895298334

H 8.53231715117691 6.44554363883960 20.13043316646377

H 9.06390460978946 4.88851112725107 19.46177224427350

C 6.10263652283174 6.58337321981786 18.79594295644747

H 5.53932448446894 6.85885726605922 17.90244978543966

H 6.36543609052190 7.50237470693557 19.32859752801186

H 5.44919821803721 5.98936813227375 19.44226547603872

C 8.38192034252765 8.97258380508799 14.45458825806574

C 8.30923584832208 10.01553908773610 15.38981658238512

C 9.00433892662960 11.18901448573870 15.12232779400385

H 8.96440982135459 12.00793695394789 15.83175676421311

C 9.75919080071179 11.32505970281342 13.96871942283069

H 10.29893215011485 12.24638067302571 13.77715782116798

C 9.82373641883345 10.28191108251782 13.06046338078462

H 10.40622295264841 10.40131577217002 12.15390439928920

C 9.13849402593319 9.09008211178344 13.28475837798806

C 7.48208839836083 9.90304247972728 16.65927719024815

H 7.17357129808492 8.86222512627663 16.77102017467453

C 6.21820596986771 10.76425569501301 16.55046641193759

H 6.48003025817800 11.82176292967090 16.44823685452414

H 5.60035443503485 10.65264759088934 17.44594739704107

H 5.61468424796826 10.48617572861174 15.68419894688045

C 8.27711810555173 10.28102627353753 17.91140271139868

H 9.17376427723079 9.67142425603660 18.01119879424753

H 7.65975421668891 10.13041512966990 18.80184700255665

H 8.58072096385765 11.33123048018329 17.89828784593522

C 9.18116203438442 7.99169586750705 12.23918905049008

H 8.71705616730511 7.10100460989525 12.66573669288381

C 10.61730034998486 7.63531498397498 11.85518827368112

H 11.14506335371342 8.49204713606482 11.43083527315907

H 10.62524464366158 6.84227415422978 11.10695673247744

H 11.18096636850903 7.28799200585966 12.72387444891821

C 8.36242879875916 8.40154949493418 11.00988162966244

H 7.32997851795101 8.63025546801255 11.28598861248581

H 8.34885136722217 7.59595337172693 10.27048013318893

H 8.78838253528160 9.29088436781575 10.53583770552036

Complex **5**

Sn 9.08775324353226 9.52584827008319 5.92332797294894

B 9.25671862125425 11.59650675487030 5.12486506549202

B 8.72210718384038 7.56442207973523 4.93556727199070

N 10.34146084540573 12.27871636792608 4.48414641145152

N 8.33068135181175 12.63894268374664 5.45118413901736

N 9.65585933236554 6.48362927626596 5.04997267787023

N 7.55487291168128 6.96016021581627 4.36536212828296

C 10.05019386386389 13.64446111460328 4.44889992834295

C 8.85487242013602 13.85912182820528 5.02646513302753

C 11.54979503782501 11.79575986964193 3.89739249858971

C 11.64823273714501 11.75208772008593 2.50188663907705

C 12.86261471213134 11.37165686014197 1.93823069987774

C 13.93896341931262 11.02529741875617 2.73644167578932

C 13.81378177889424 11.04839148852374 4.11713436062360

C 12.62498943466827 11.44223568307608 4.72370281591651

C 10.48457591376694 12.12909174245808 1.60225488906103

C 10.16515671152476 11.01999693375896 0.59656994322438

C 10.75791088762255 13.45109915505452 0.87740776894432

C 12.53226954752671 11.56474839066643 6.23351524601251

C 13.31872081244317 10.48281407722199 6.97332792254384

C 12.97541522154153 12.96443348786541 6.67501964163753

C 7.06507863225929 12.56003594971637 6.10667982954967

C 7.01974303711016 12.48554879332780 7.50334219162600

C 5.77013610333589 12.42766499970092 8.11305167388509

C 4.60858752419199 12.44113521678645 7.35837655117568

C 4.67555364232147 12.51133943542582 5.97578109546536

C 5.90380537359646 12.57593322534529 5.32513652636947

C 8.29060373066231 12.51845491775586 8.33323065301060

C 8.19962743215607 11.68635399910639 9.61298707690599

C 8.67105137010169 13.96812933751115 8.65788721108370

C 5.99601861867614 12.64037755950985 3.80969553899259

C 4.76507332494060 13.27060714476257 3.15630605646050

C 6.24700218806843 11.24625528336719 3.22486284708420

C 9.05793303321961 5.31610637137494 4.57922606193192

C 7.80844510151044 5.59998313049775 4.17002359426127

C 10.97639504358686 6.48793186689397 5.59282932696114

C 12.06541500149928 6.57066428282456 4.71813842727781

C 13.34467030533188 6.58828478081718 5.26595938963049

C 13.53037924124232 6.52170882763978 6.63776165169218

C 12.43803619004067 6.43895551840983 7.48584625476184

C 11.14184284068192 6.42145777362978 6.98063063576073

C 11.84701202840699 6.67478573572217 3.21835569311506

C 11.57512406860373 8.12850517679298 2.81712597567774

C 13.01094881358645 6.11457670631175 2.39851701343617

C 9.94713797375598 6.30297500666322 7.90940578428335

C 10.14480139218595 7.03209466376736 9.23915350926771

C 9.60253071971977 4.82747870500828 8.14569558347561

C 6.29514103724442 7.50570442276708 3.97282956459679

C 5.34067319184370 7.81951230907092 4.94952094458229

C 4.09496958694852 8.27291342195732 4.52542315398251

C 3.79748056422324 8.39301403467832 3.17692791498794

C 4.75750789694331 8.08650648460742 2.22778325118679

C 6.02303670521048 7.64901621773585 2.60677866165568

C 5.61070527053442 7.59436089801220 6.42573219563527

C 5.02094497831397 8.69013817566112 7.31338393662333

C 5.10190559817478 6.21004197912221 6.84410308985990

C 7.06049304841261 7.33716106404681 1.54230237657237

C 6.65532872784606 6.11423156199673 0.71337769626479

C 7.30875346081604 8.54753697632259 0.63822870614151

H 7.89613986959143 9.63469028230743 7.14820096136679

H 10.53016405823906 9.28065444795771 6.81027186661474

H 10.73551011457708 14.35427640245564 4.01318881197706

H 8.32287097372346 14.78661668499877 5.17260593651028

H 12.96550932781294 11.34127416546843 0.85890131400457

H 14.87861500530575 10.72855944190063 2.28232745524511

H 14.66082434415791 10.76798478410731 4.73176102741772

H 9.60281940485797 12.26555823805117 2.23097129192450

H 11.48534828172839 11.45086677102399 6.51230667581737

H 13.05613565991742 9.48404213753953 6.61852867784688

H 14.39876810026903 10.61622257576189 6.86306478905738

H 13.09464702168190 10.53094434099974 8.04242931765353

H 12.87471927631700 13.07315469852512 7.75896203462554

H 14.02319820777707 13.13835038828805 6.41156822658405

H 12.37067829790410 13.73946973223010 6.19882668917328

H 5.70079381342021 12.36944976712596 9.19266231953715

H 3.64330121082601 12.39243067199072 7.85148536476866

H 3.75834646457346 12.52000890783591 5.39987837072395

H 9.09338315276673 12.09481039721535 7.72621480742040

H 9.18323606051133 11.63103635950183 10.08701635012655

H 7.51464546788162 12.13050942115795 10.34045102726276

H 7.86557978841983 10.66850224894155 9.40022575968076

H 9.60236092612622 14.00230218629180 9.23061051519829

H 8.81115199154034 14.55531933213137 7.74831587483542

H 7.88629486570772 14.44404222445192 9.25393706843308

H 6.85527422549331 13.26727565864182 3.55884166684452

H 4.95258149823618 13.42853659792952 2.09105692195942

H 4.51873866695773 14.23617480956426 3.60555806080650

H 3.88790258008713 12.62253299775342 3.23727507212318

H 6.31421441195771 11.29708871249288 2.13440455147824

H 7.17562433691628 10.81314258003552 3.59949799299288

H 5.43197103755118 10.56784061033641 3.48313468323205

H 9.58104017806028 4.37222998215674 4.57448986137327

H 7.06162956627480 4.94471872420821 3.75007460317493

H 14.20896132065196 6.65237396774807 4.61637349949193

H 14.53432917319688 6.53719570681479 7.04898631022296

H 12.59914950037790 6.39229958898775 8.55619143203964

H 10.95856896635434 6.08746020221769 2.97259461781212

H 11.43505817845622 8.20590466998135 1.73521706647044

H 12.41351370615845 8.76930920079275 3.09600435178327

H 10.67903068879856 8.51823718406689 3.30241337509948

H 12.73394422718567 6.07588456264524 1.34180004731177

H 13.89967859733577 6.74704356040328 2.47505576404174

H 13.28227288029357 5.10480463211410 2.71714735299509

H 9.09148859688808 6.76284032775140 7.41035579269366

H 9.20384931514560 7.04666120855885 9.79574048564680

H 10.88624580591923 6.53439220965355 9.87014606373121

H 10.46410748161071 8.06460910820986 9.08037777564231

H 8.72356594280484 4.73623562985311 8.79042927873826

H 10.43709560912436 4.31295653951639 8.63183889389096

H 9.38755676281369 4.31512498631820 7.20596643562296

H 3.33854225408290 8.52071415028996 5.26071612333375

H 2.81556006715763 8.73442011694377 2.86619057616851

H 4.52201475984578 8.19578989485293 1.17458174053647

H 6.68983576563011 7.60414832342314 6.57335461523732

H 5.36202868591019 8.55588991428674 8.34356476425370

H 5.33126526629369 9.68265334068989 6.98342699572666

H 3.92768896305894 8.65977020334430 7.32735543784642

H 5.32802389344699 6.02086544778433 7.89760300911602

H 5.56899957785869 5.42175679118796 6.24868053162623

H 4.01787447272076 6.14051757583696 6.71140414830331

H 8.00081780683162 7.10521730807147 2.04505761174331

H 6.50653541892899 5.23566824322024 1.34524799481740

H 5.72223435142436 6.29799000332995 0.17263497645908

H 7.42901136096233 5.87706904337910 -0.02239623505848

H 8.11064560848386 8.33218875911674 -0.07360848345845

H 7.59831552478090 9.42072166003971 1.22475576007154

H 6.41545719196854 8.80797731777844 0.06378491242371

H 9.27226203008962 11.27749146189257 0.01998900159271

H 10.98457906795475 10.87131899546666 -0.11207572067791

H 9.98071559659543 10.07142978811444 1.10307098096067

H 9.90021757728011 13.73455464715538 0.26074088059148

H 10.95021017714452 14.26166079429046 1.58350509113348

H 11.62972085566548 13.36358227933935 0.22231832127363

Complex **6**

Sn 4.06257819518198 8.77942703532680 15.03947198623333

B 4.93224952408847 10.74740063938476 14.44478493293802

B 4.43164819358452 7.28612060266539 16.64296641138398

Cl 1.75596412874067 9.16498019266704 15.32051942097069

Cl 4.08242581803613 7.57449715377397 13.01113338070051

N 4.17847344975008 11.94864860408211 14.26052092637441

N 6.27867302139241 11.09138438543188 14.09574435056811

N 4.96353830058918 5.96934168743812 16.51526800571152

N 4.12632057243863 7.43962495879622 18.03013917614357

C 5.05526569186292 12.95029873091728 13.84732700946330

C 6.29626728286246 12.44575302060489 13.75253619023640

C 2.77072960472839 12.22177242714387 14.28284389774901

C 1.97119173756504 11.74898758227169 13.23281582315856

C 0.62339093531274 12.08300734901718 13.24050882417159

C 0.08253618754425 12.85825178333553 14.25503880008949

C 0.88807498517433 13.30715235609291 15.28434268588073

C 2.24482692362316 12.99515661551326 15.31822312054433

C 2.54945309177325 10.91799109945081 12.10200137666398

C 3.11321455502978 11.82503545540176 11.00187464175920

C 1.55352215413657 9.91615790335491 11.51841218913977

C 3.09825033968894 13.50585108540000 16.46269346454228

C 3.27665027440374 15.02557196669107 16.38186210702673

C 2.51086738938971 13.09545791895942 17.81521983654448

C 7.47630344492766 10.33513616692624 13.86644644159535

C 8.50409003816866 10.37778294038960 14.81446053110353

C 9.72061100564101 9.78003726254262 14.49808691334200

C 9.90614557171118 9.13816626951426 13.28787848598773

C 8.86989812854522 9.08484240369487 12.37027872705492

C 7.64395318339283 9.68857314619506 12.63162994083821

C 8.35532020411529 11.10122537988924 16.13955127209006

C 9.18275149266199 12.39168671699980 16.13917655339108

C 8.74943516234411 10.21067192460631 17.32223655673668

C 6.57927202432162 9.71934560179819 11.54966612672157

C 6.81583396425623 10.91992665102756 10.62326077526402

C 6.52454358280946 8.42843968538979 10.72935799458102

C 5.00028512729708 5.39462153818133 17.78650874192155

C 4.50810729528448 6.26624833188949 18.68393589194329

C 5.28051363524639 5.19302173798849 15.35417355194932

C 6.62372021198732 4.96650693046495 15.04200031452798

C 6.91597185127949 4.20306906031500 13.91630970314501

C 5.89922693684520 3.67297983601846 13.13920413391962

C 4.57436807801133 3.89668843223876 13.47654842039834

C 4.23589513453689 4.66082152399652 14.58808196792792

C 7.72572496966756 5.58224407128960 15.88419252431868

C 9.03360580396177 4.79026160698335 15.85523696629756

C 2.77993265439267 4.88426717275017 14.96234558916169

C 1.82381648248972 4.81534353135660 13.77166195750697

C 2.34119940074592 3.88764203728551 16.04371305236370

C 3.30493048241797 8.39000689552767 18.71767253278317

C 1.94242684473806 8.09374182083465 18.88283536214314

C 1.17042258670251 8.97255732032399 19.63324852934428

C 1.72893021126301 10.10301768653828 20.20840801764153

C 3.07042232497349 10.38280133060954 20.02079960656741

C 3.88014724039961 9.53993688317586 19.26349475898458

C 5.35352822429423 9.86044619836311 19.09279623497109

C 5.57076908020180 11.31474253355228 18.67031449792666

C 6.12335320914786 9.54005223576656 20.37801844031192

C 1.31411261153508 6.83312813698660 18.31315520129492

C -0.05447932185240 7.08328936898783 17.67350419581067

C 1.17766744701052 5.75773949126641 19.39887492538359

C 7.97124194483838 7.02650586524608 15.44459149848999

H 4.70650997853311 13.95011877529198 13.64312009884979

H 7.21036559114598 12.93182221224624 13.45074069435902

H -0.02078173662613 11.72687662909539 12.44627643239169

H -0.97319257797294 13.10799737404546 14.24228571239868

H 0.45769664296573 13.90793218499033 16.07787258112993

H 3.37954286104271 10.33478384012013 12.50063695243889

H 3.53336051748049 11.22333362769529 10.19103947968856

H 3.90168666071747 12.47696985841951 11.38322389751990

H 2.32123225935285 12.45384999760183 10.58349953750548

H 0.76693579287833 10.41343762160133 10.94343610458535

H 1.08898698655288 9.31828838363893 12.30487574753706

H 2.07577632134814 9.23605350533804 10.84134619847708

H 4.08641469857811 13.05078853089346 16.37500677672982

H 3.73776116724680 15.32470318332901 15.43757519646882

H 2.31195492376090 15.53544713649579 16.45709218402860

H 3.91174702709383 15.38199125134933 17.19799712909404

H 3.18088394644156 13.39316884589188 18.62629682397934

H 1.54469728953145 13.57508869838787 17.99306991130658

H 2.36389521728703 12.01562888371850 17.87056662985380

H 10.53705736672520 9.82034387912071 15.21040425315096

H 10.86086179286337 8.67763822009442 13.05669907408974

H 9.02464181246348 8.58161550896437 11.42369076808603

H 7.30532393486018 11.37411733468390 16.25732671044064

H 9.04426729002241 12.93805441099169 17.07654074395372

H 10.24832243270170 12.16944004717126 16.03078747726788

H 8.89315911524140 13.04958694914814 15.31699609952012

H 8.67392286870486 10.76699203345928 18.25968532116382

H 9.78168395905826 9.86245383389691 17.23801508698645

H 8.10386980856854 9.33329620247870 17.39384477338442

H 5.60787898244316 9.84381054854125 12.02837558872799

H 6.06176748076982 10.94532640602415 9.83222492496339

H 6.76737799532733 11.86540238778778 11.16547043387033

H 7.80039651065408 10.84548695607977 10.15141514890183

H 6.48933293695749 7.54585544001021 11.36950525011221

H 7.38451825412270 8.34222362439330 10.05894880026887

H 5.62595896179285 8.42738071042475 10.10744802359702

H 5.38136591852252 4.39673275090813 17.93964466126972

H 4.38999683355517 6.15603396135899 19.75010433886973

H 7.94742871101905 4.01667980885398 13.64319048714751

H 6.14094692566290 3.07866112224735 12.26420873373193

H 3.79246428926846 3.47749121171396 12.85605102707549

H 8.87215358510911 3.73455564297306 16.08760579573899

H 9.72828602454065 5.19949969579959 16.59353332309794

H 9.52471415991377 4.85385457962767 14.88025230979011

H 2.69247919397146 5.89000430772475 15.37947156189224

H 0.83088761041168 5.14828049126398 14.08475395236130

H 2.15557894692693 5.45848046319886 12.95576494878976

H 1.72082696476938 3.79355450395539 13.39478935501453

H 2.41901916670804 2.86314280816350 15.66724569576640

H 2.95146149770781 3.96826503893214 16.94439899226483

H 1.29900427031164 4.06610714863393 16.32451126746330

H 0.11579159958411 8.76780363273047 19.77636244381275

H 1.11250008064253 10.77226216298426 20.79920277459554

H 3.49434445585784 11.27298209024231 20.47068031955678

H 5.74760385155570 9.21845073608486 18.30207815761453

H 6.63294555455834 11.51589726398145 18.52786863965806

H 5.20711825587136 12.01364057665686 19.42665298570272

H 5.05208892850848 11.53478318961838 17.73459101088321

H 7.19029340249694 9.74394113903381 20.25099231838291

H 5.75726959390709 10.14929807945728 21.20964828004101

H 6.00637434228255 8.48922990968114 20.65436333347993

H 1.97751177047446 6.45011506019004 17.53600427660846

H -0.38277894820723 6.18233689687485 17.14745919365708

H -0.81392445239305 7.30967316663420 18.42703216136807

H -0.01914397039543 7.90319326747547 16.95736534932416

H 0.74780425859023 4.84513364225124 18.97629831607858

H 0.51485238918034 6.10627615852930 20.19667843552627

H 2.13935703233632 5.50284277444671 19.84559228189665

H 7.07557000146076 7.63767067875857 15.56973868104584

H 8.77250347530905 7.47757213546971 16.03258536560418

H 8.25947504468790 7.07026888643048 14.39235065435246

H 7.38340353744749 5.60483370305703 16.92143650996802

# References

1. A. V. Protchenko, K. H. Birjkumar, D. Dange, A. D. Schwarz, D. Vidovic, C. Jones, N. Kaltsoyannis, P. Mountford, S. Aldridge, *J. Am. Chem. Soc.* **2012**, *134* (15), 6500–6503.
2. M. Arrowmsith, J. Böhnke, H. Braunschweig, A. Deißenberger, R. D. Dewhurst, W. C. Ewing, J. Mies, J. H. Muessig, *Chem. Commun.* **2017**, *53*, 8265–8267.
3. J. T. Boronski, A. E. Crumpton, L. L. Wales, S. Aldridge, *Science* **2023**, *380*, 1147–1149.
4. T. Wideman, L. G. Sneddon, *Inorg. Chem.* **1995**, *34*, 1002–1003.
5. J. Cosier, A. M. Glazer, A nitrogen-gas-stream cryostat for general X-ray diffraction studies. *J. Appl. Cryst.* **1986**, *19*, 105–107.
6. CrysAlisPro v.1.171.42.70a, Agilent Technologies, **2011**.
7. G. Sheldrick, SHELXT ‒ Integrated space-group and crystal-structure determination. *Acta Cryst.* **2015**, *A71*, 3–8.
8. G. M. Sheldrick, A short history of SHELX. *Acta Cryst.* **2008**, *A64*, 112–122.
9. G. M. Sheldrick, Crystal structure refinement with SHELXL. *Acta Cryst.* **2008**, *A64*, 112–122.
10. C. B. Hübschle, G. M. Sheldrick, B. Dittrich, ShelXle: a Qt graphical user interface for SHELXL. *J. Appl. Cryst.* **2011**, *44*, 1281–1284.
11. F. Neese, F. Wennmohs, U. Becker, C. Riplinger, *J. Chem. Phys.* **2020**, *152*, 224108.
12. F. Neese, *WIREs Comput. Mol. Sci.* **2012**, *2*, 73–78.
13. Y.-S. Lin, G.-D. Li, S.-P. Mao, J.-D. Chai, *J. Chem. Theory Comput.* **2013**, *9*, 263–272.
14. E. Caldeweyher, J.-M. Mewes, S. Ehlert, S. Grimme, *Phys. Chem. Chem. Phys.* **2020**, *22*, 8499–8512.
15. A. Canal Neto, I. B. Ferreira, F. E. Jorge, A. Z. de Oliveira, *Chem. Phys. Lett.* **2021**, *771*, 138548.
16. N. Mardirossian, M. Head-Gordon, *Phys. Chem. Chem. Phys.* **2014**, *16*, 9904.
17. E. D. Glendening, J. K. Badenhoop, A. E. Reed, J. E. Carpenter, J. A. Bohmann, C. M. Morales, P. Karafiloglou, C. R. Landis, F. Weinhold, Theoretical Chemistry Institute, University of Wisconsin, Madison, WI, **2018**.
18. T. Lu, F. Chen, *J. Comput. Chem.* **2012**, *33*, 580–592.
